# Supplementary figures and images for: BAG3 regulates bone marrow mesenchymal stem cell proliferation by targeting INTS7
Source: PeerJ. 2023 Aug 9;11:e15828. doi: 10.7717/peerj.15828 (PMC10422954; doi:10.7717/peerj.15828)

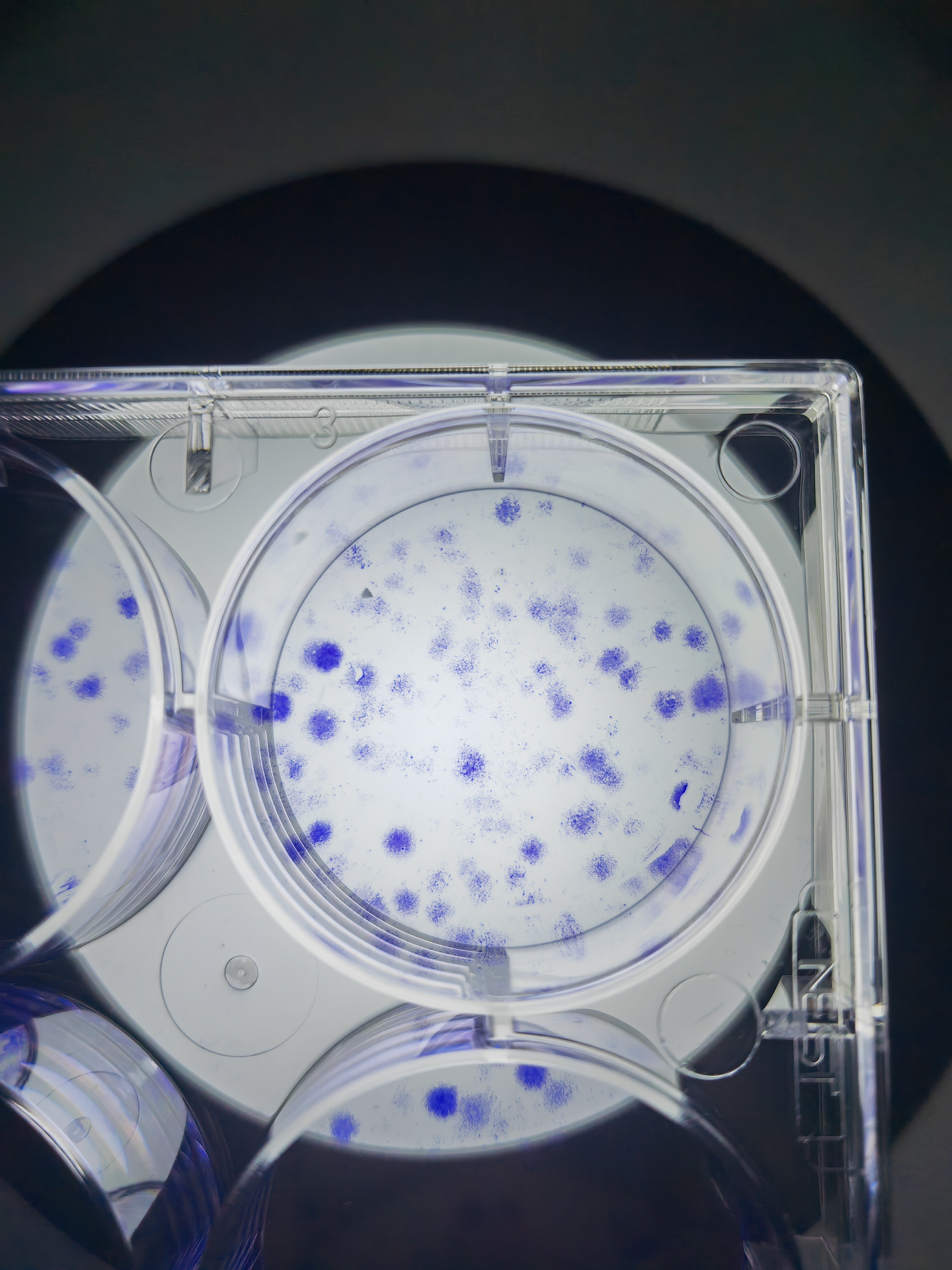

Supplement: Supplemental Information 1 [file peerj-11-15828-s001.zip › The raw data of colony formation/The raw data of clone/figure1e/nc.jpg]

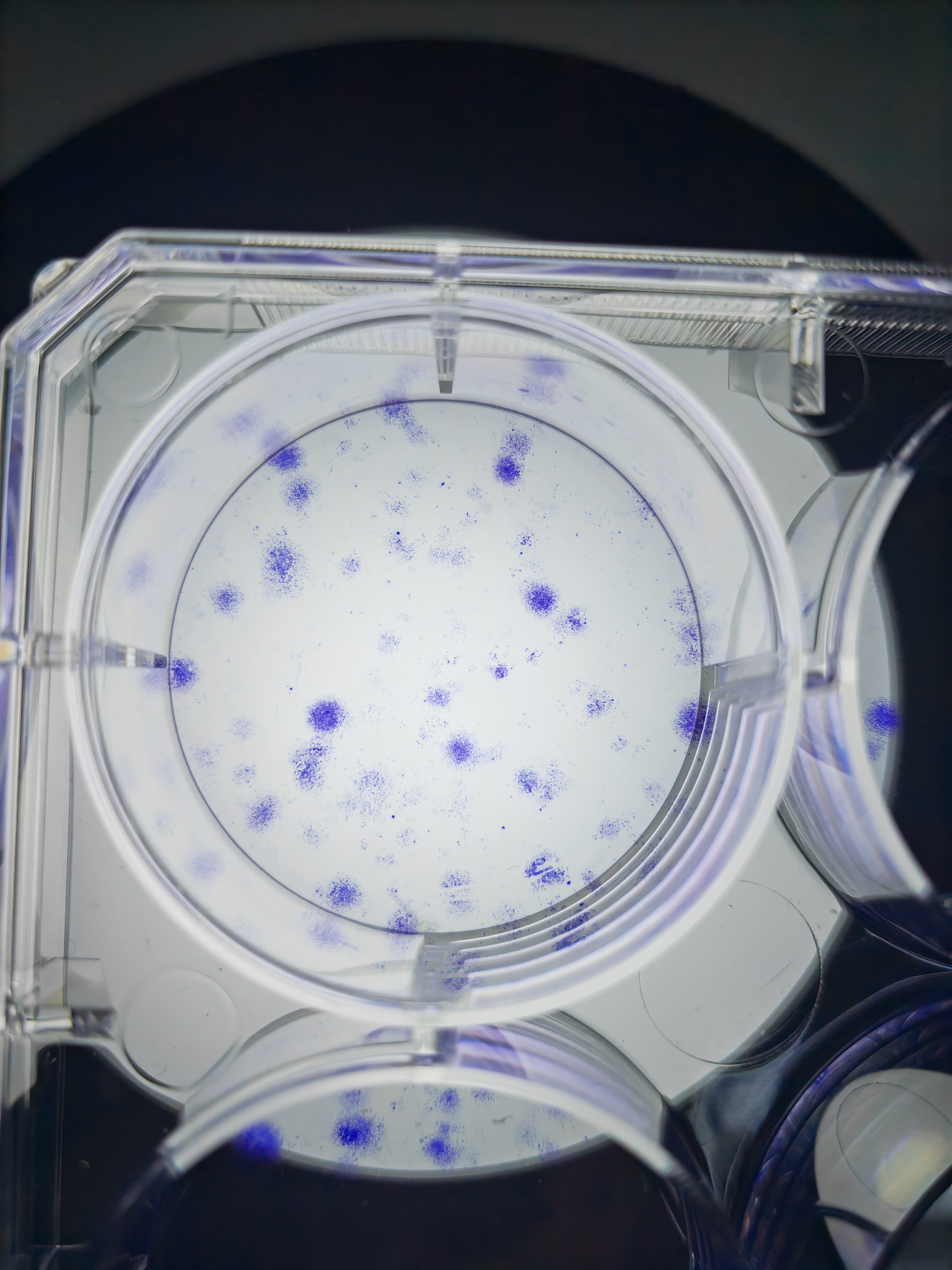

Supplement: Supplemental Information 1 [file peerj-11-15828-s001.zip › The raw data of colony formation/The raw data of clone/figure1e/si-bag3-1.jpg]

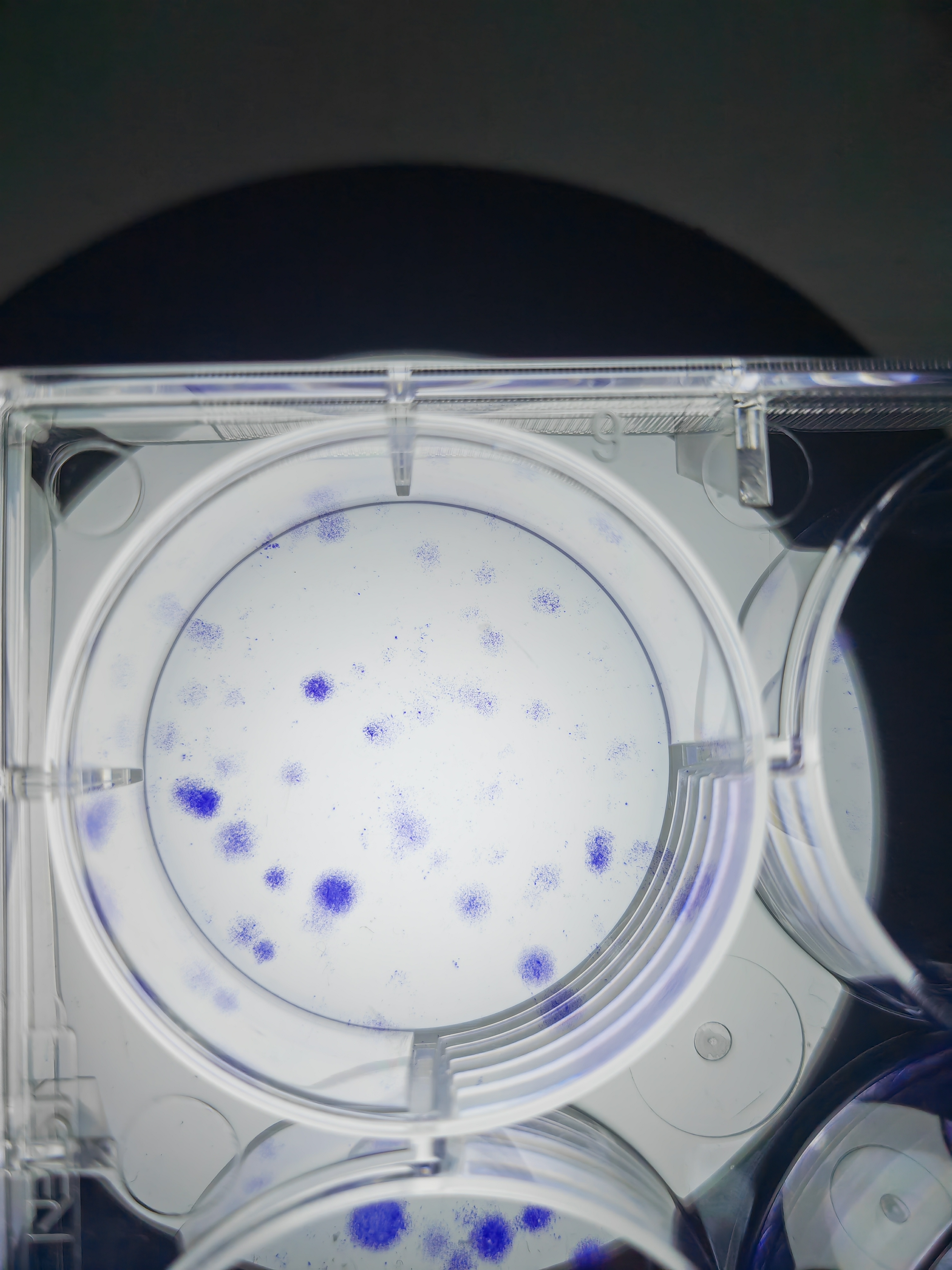

Supplement: Supplemental Information 1 [file peerj-11-15828-s001.zip › The raw data of colony formation/The raw data of clone/figure1e/si-bag3-2.jpg]

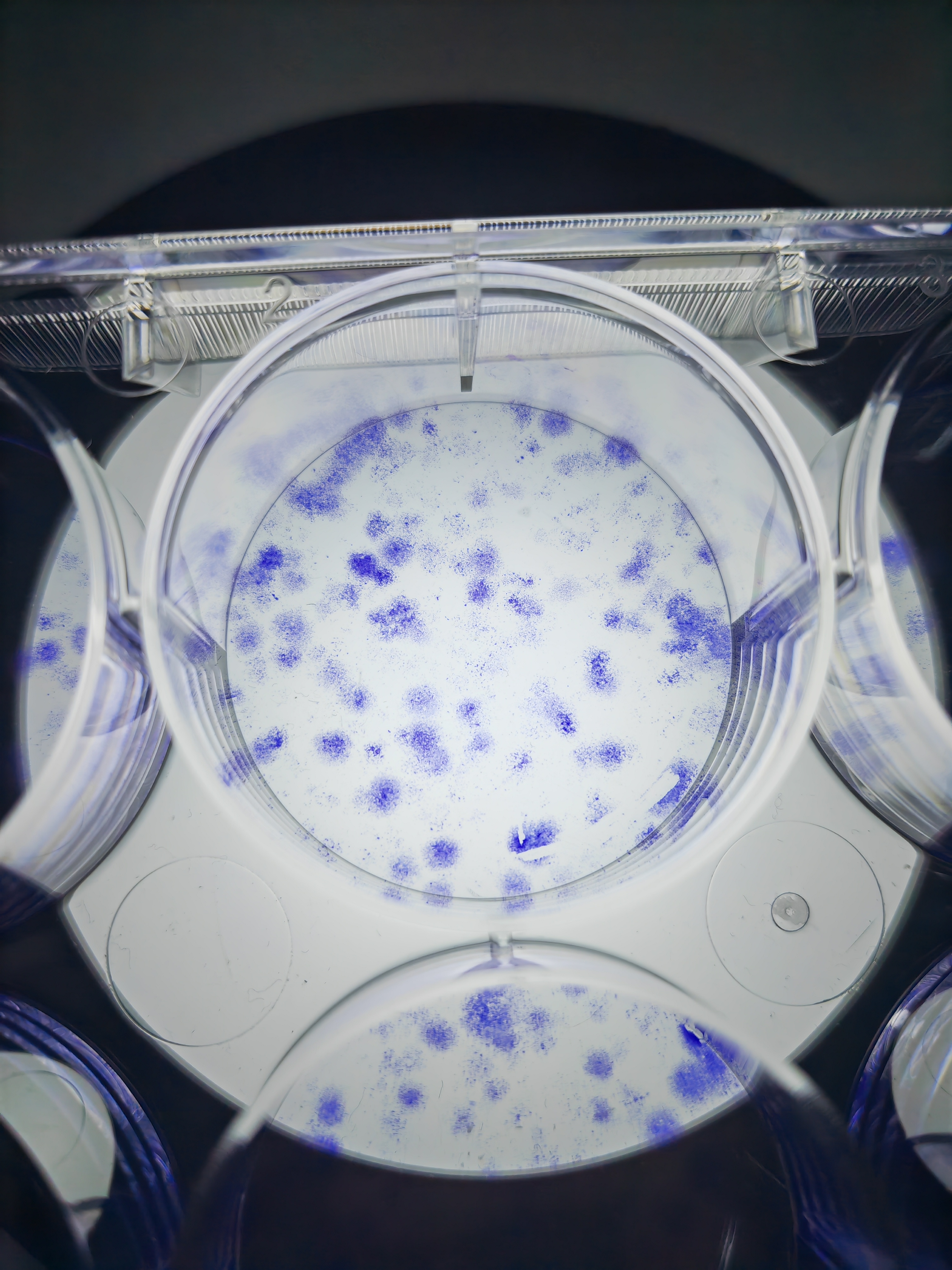

Supplement: Supplemental Information 1 [file peerj-11-15828-s001.zip › The raw data of colony formation/The raw data of clone/figure2d/bag3.jpg]

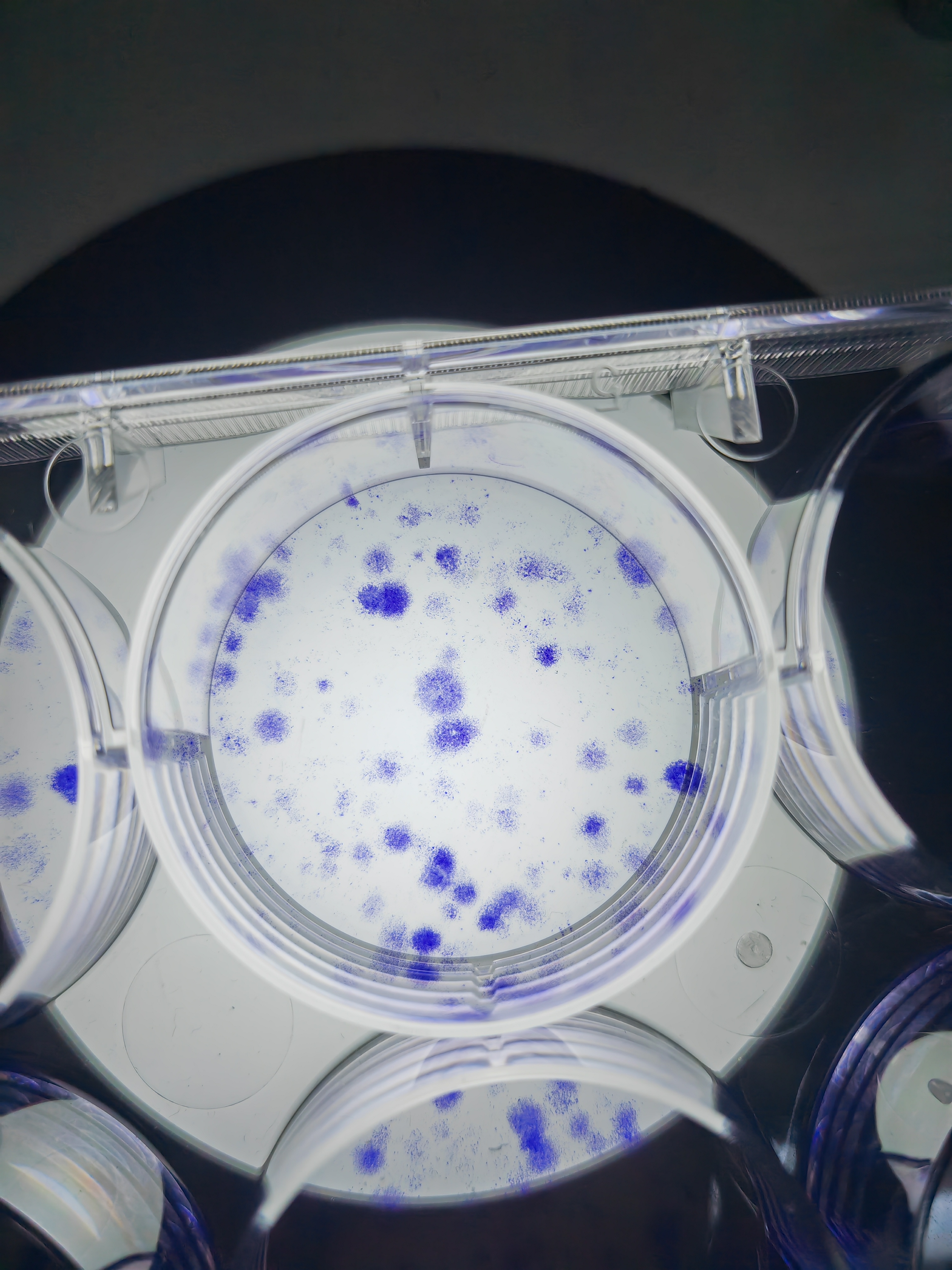

Supplement: Supplemental Information 1 [file peerj-11-15828-s001.zip › The raw data of colony formation/The raw data of clone/figure2d/nc.jpg]

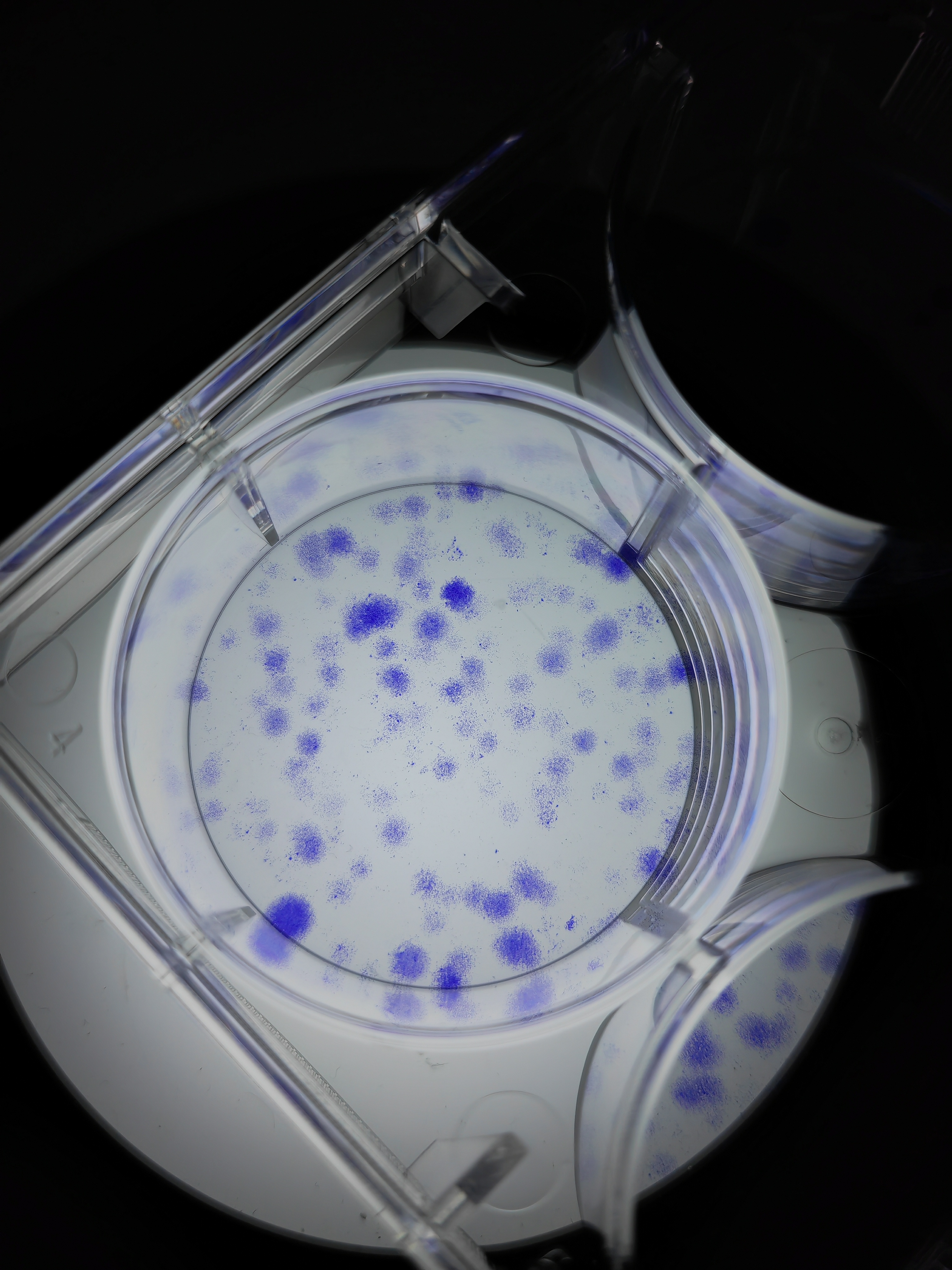

Supplement: Supplemental Information 1 [file peerj-11-15828-s001.zip › The raw data of colony formation/The raw data of clone/figure5b/nc.jpg]

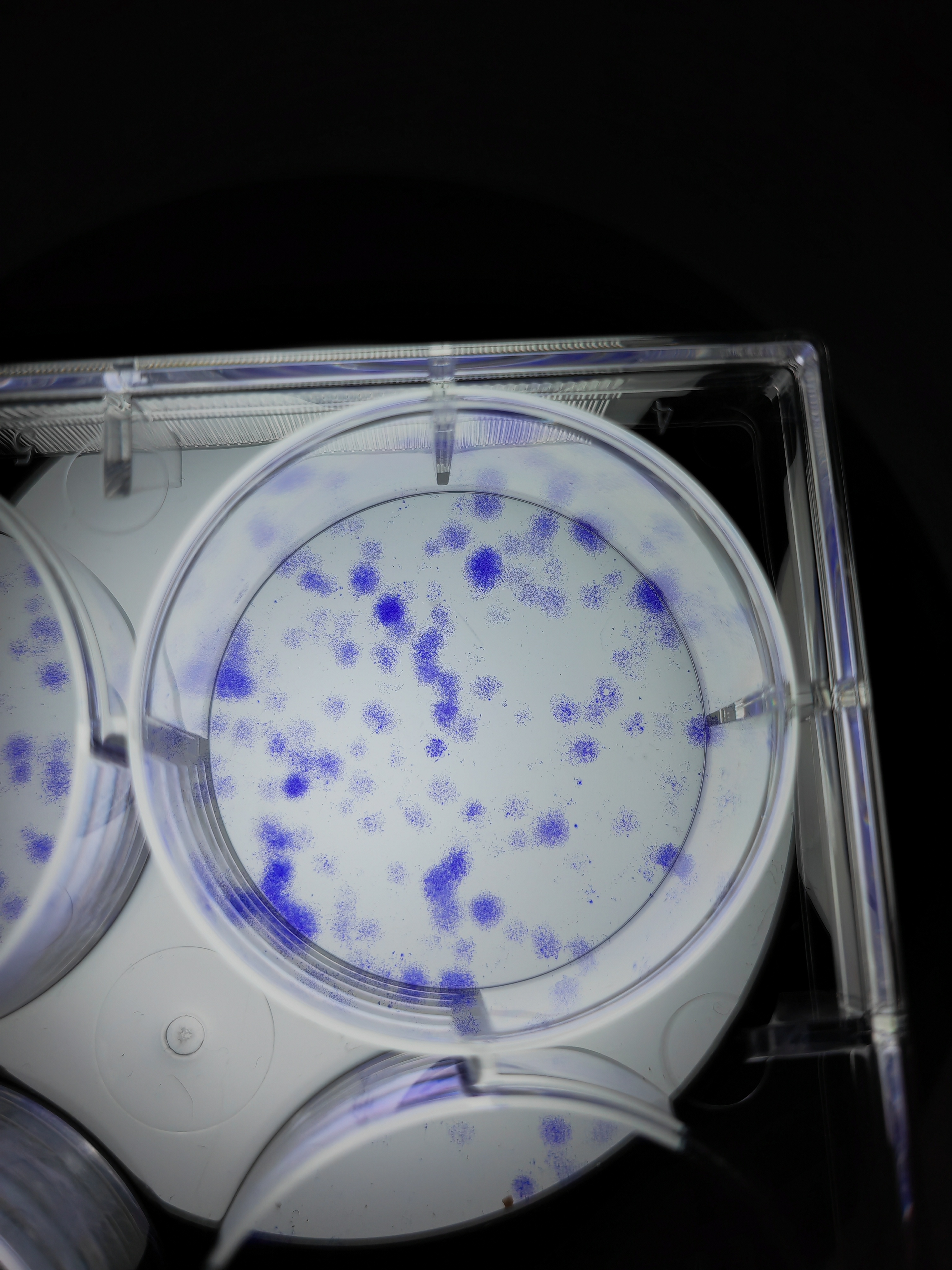

Supplement: Supplemental Information 1 [file peerj-11-15828-s001.zip › The raw data of colony formation/The raw data of clone/figure5b/si-bag3+ints7.jpg]

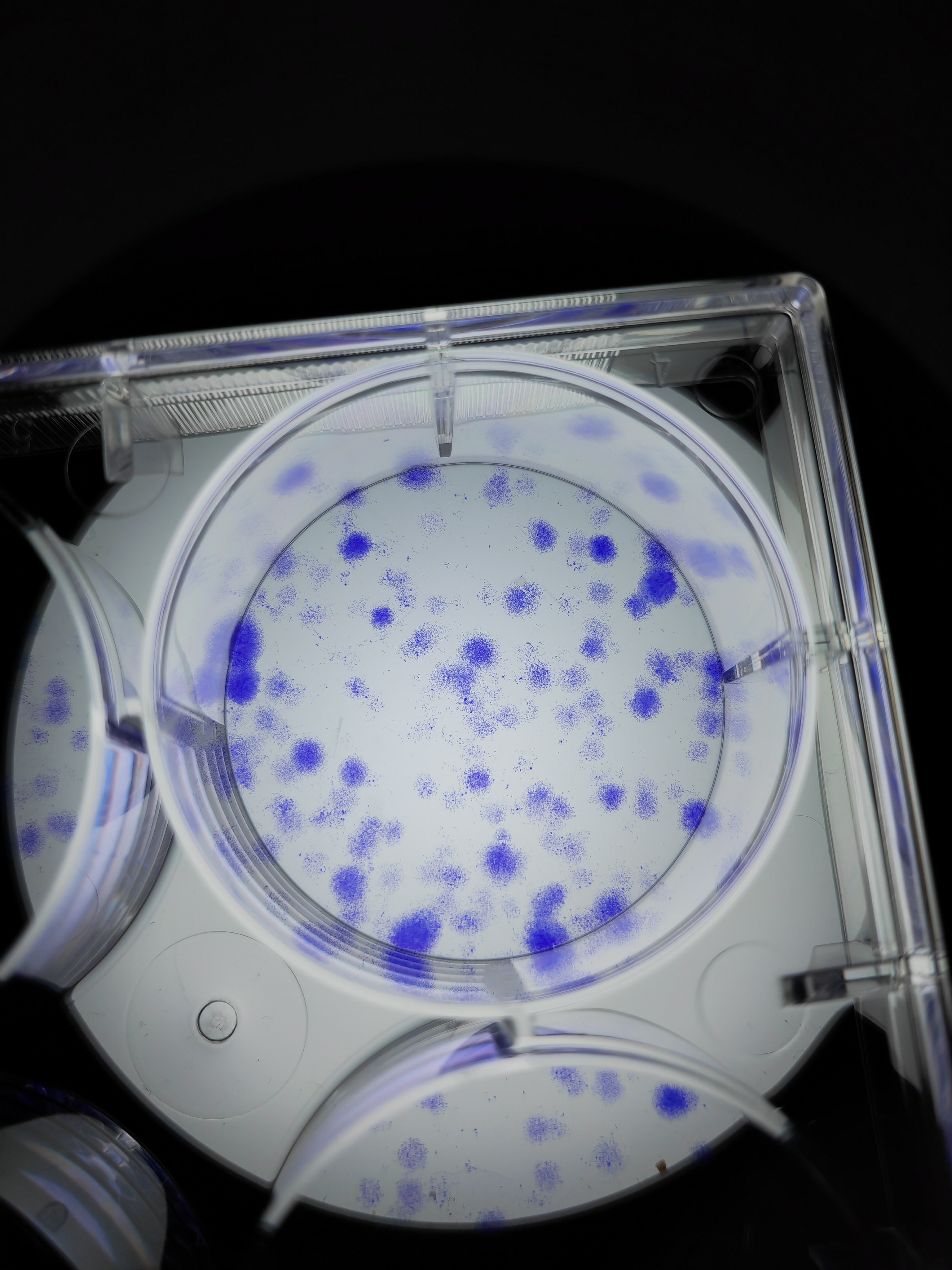

Supplement: Supplemental Information 1 [file peerj-11-15828-s001.zip › The raw data of colony formation/The raw data of clone/figure5b/si-bag3+nac.jpg]

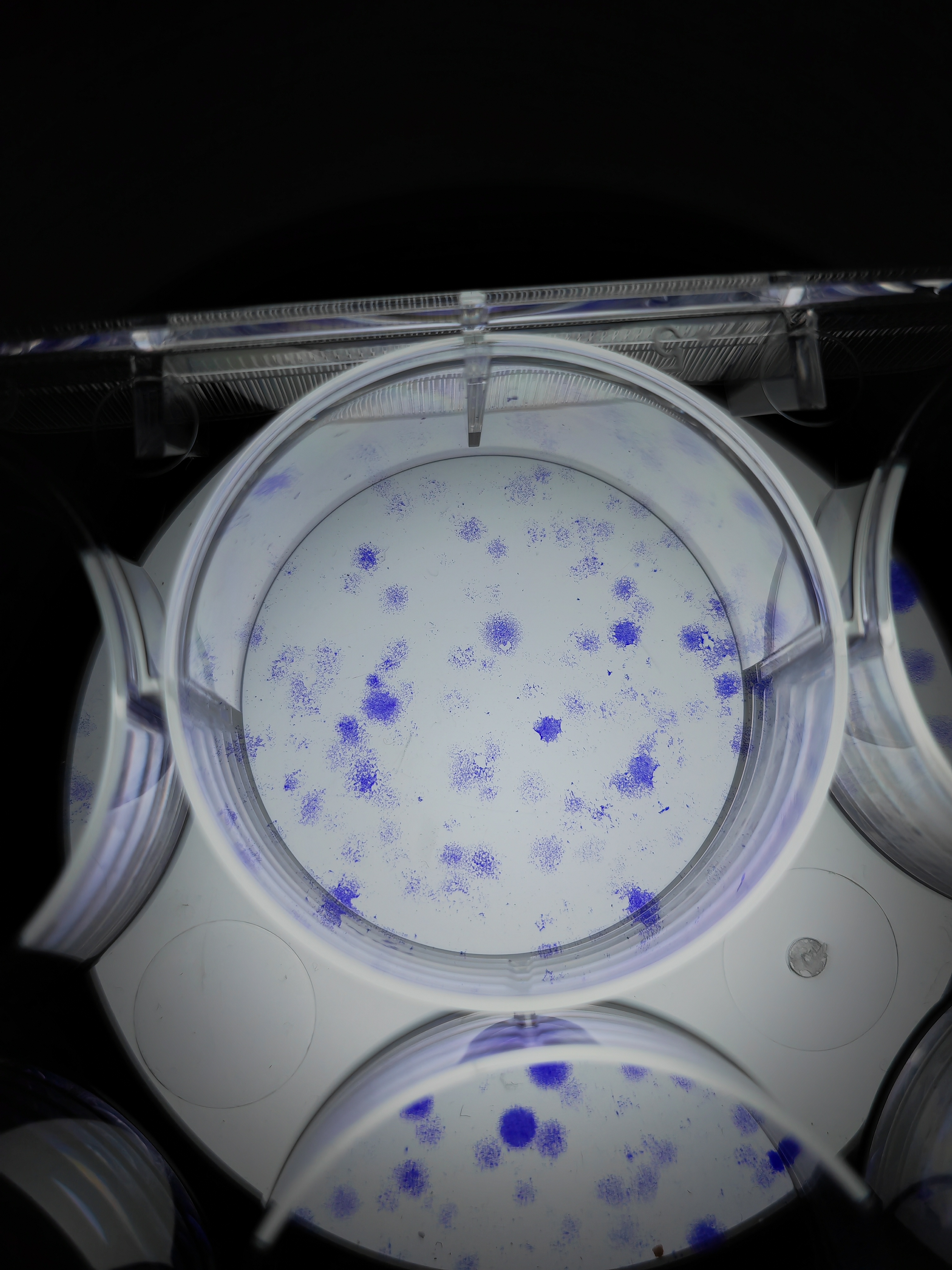

Supplement: Supplemental Information 1 [file peerj-11-15828-s001.zip › The raw data of colony formation/The raw data of clone/figure5b/si-bag3.jpg]

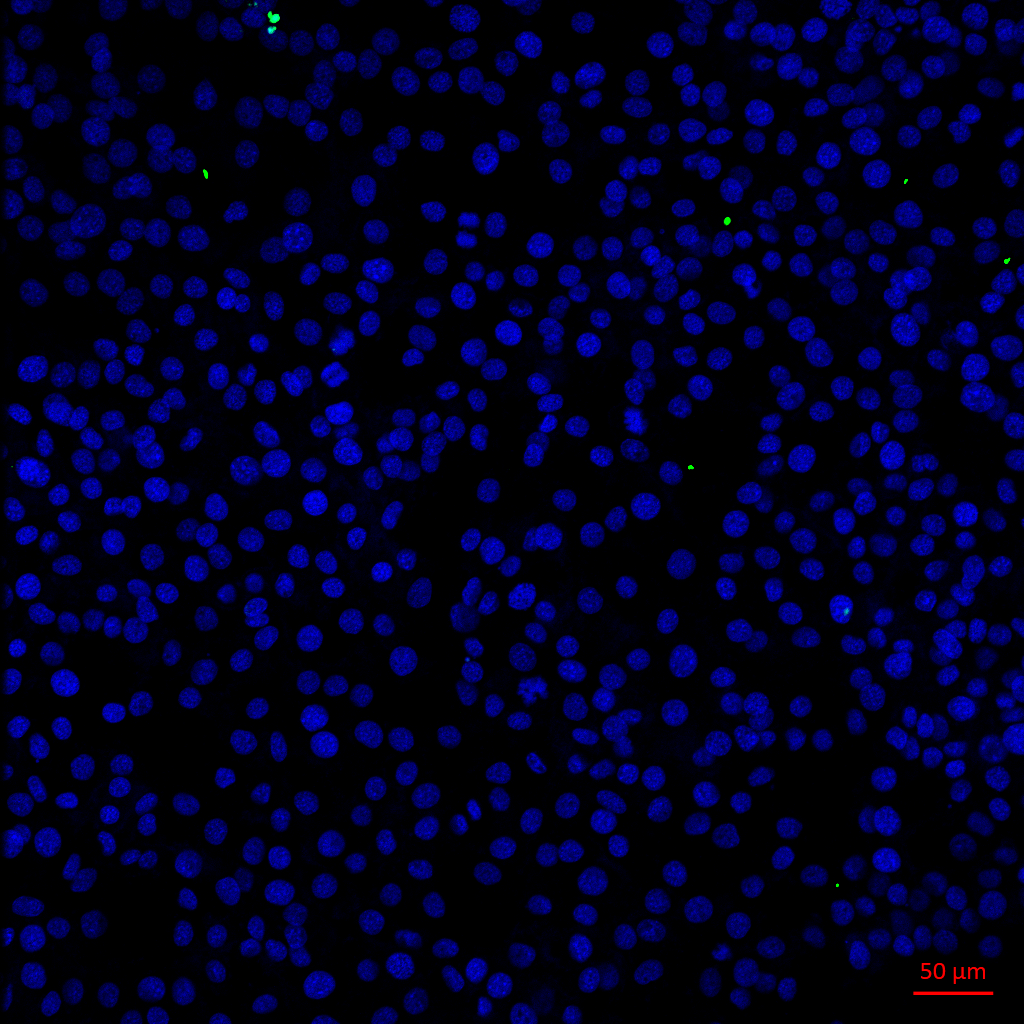

Supplement: Supplemental Information 2 [file peerj-11-15828-s002.zip › The raw data of immunofluorescence/figure-4c-nc/Image 39nc_c1+2.tif]

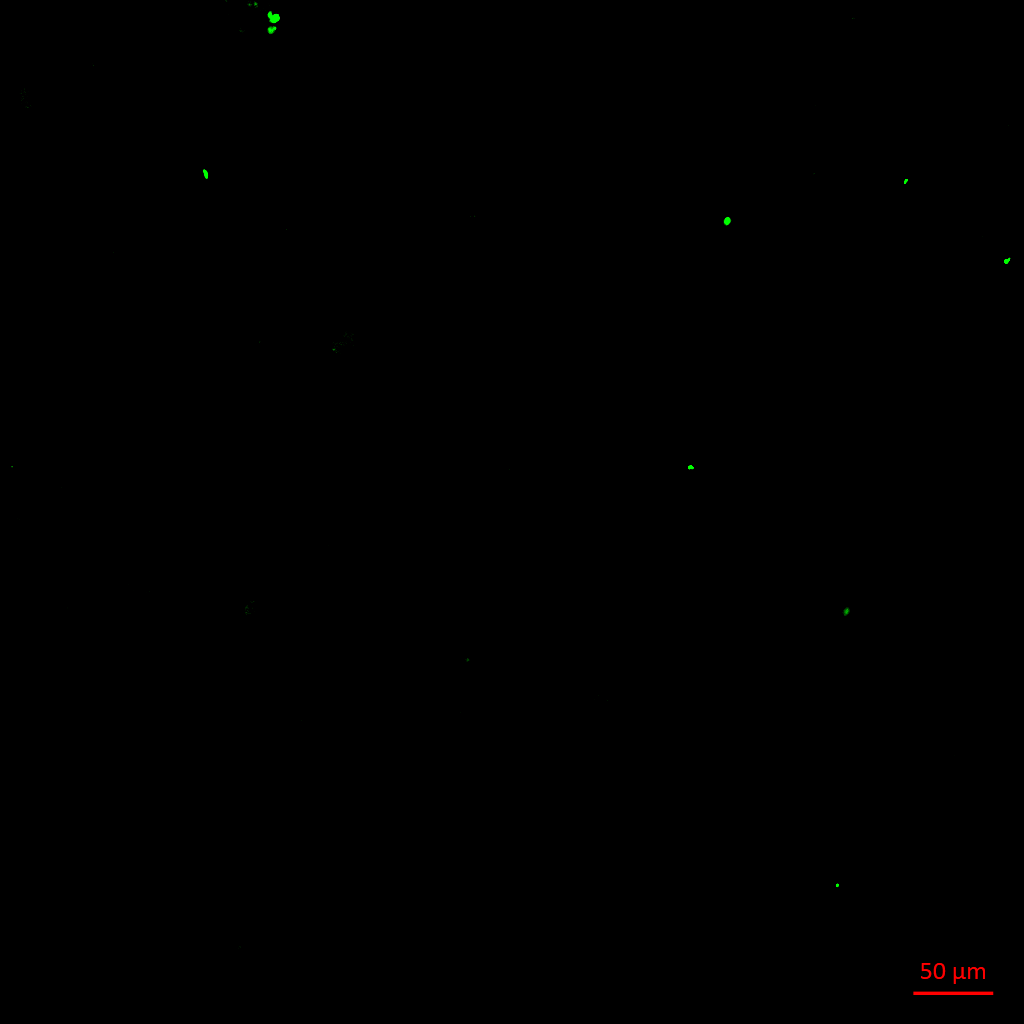

Supplement: Supplemental Information 2 [file peerj-11-15828-s002.zip › The raw data of immunofluorescence/figure-4c-nc/Image 39nc_c1.tif]

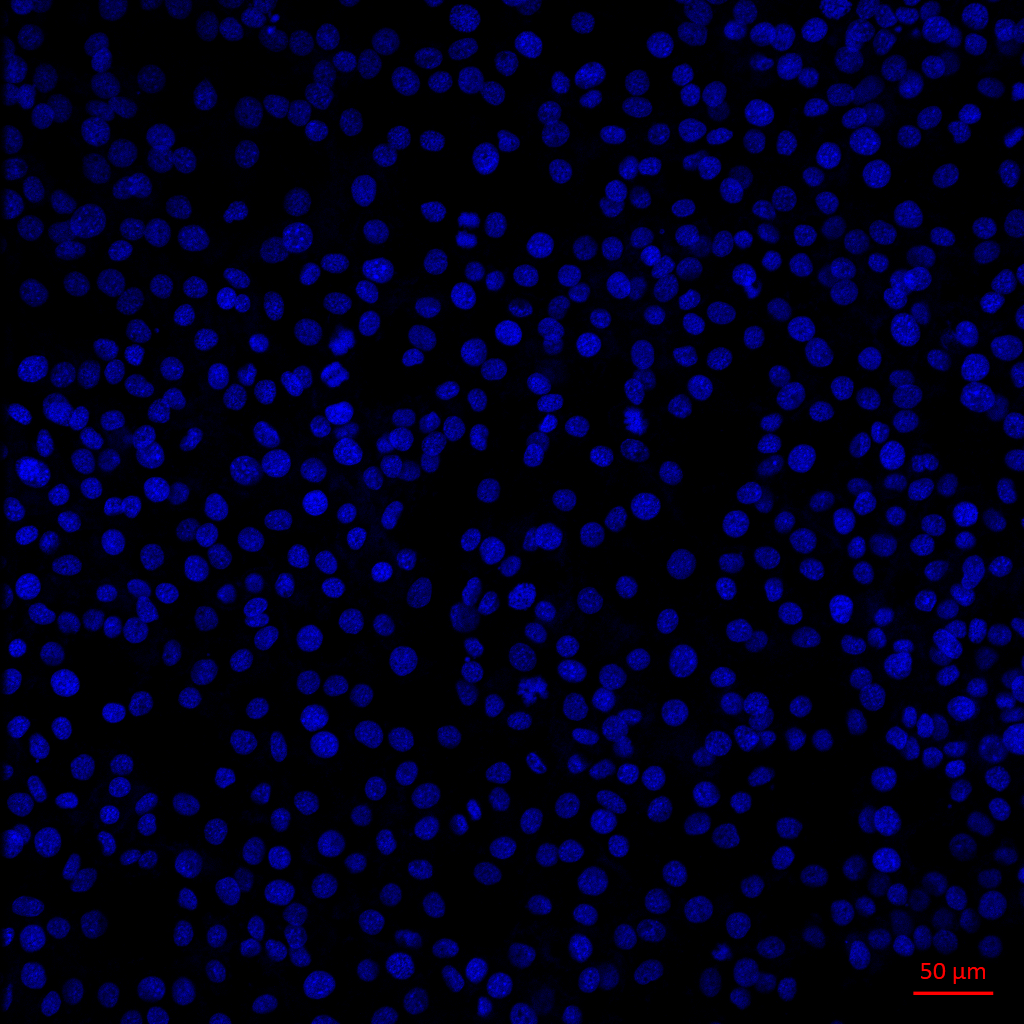

Supplement: Supplemental Information 2 [file peerj-11-15828-s002.zip › The raw data of immunofluorescence/figure-4c-nc/Image 39nc_c2.tif]

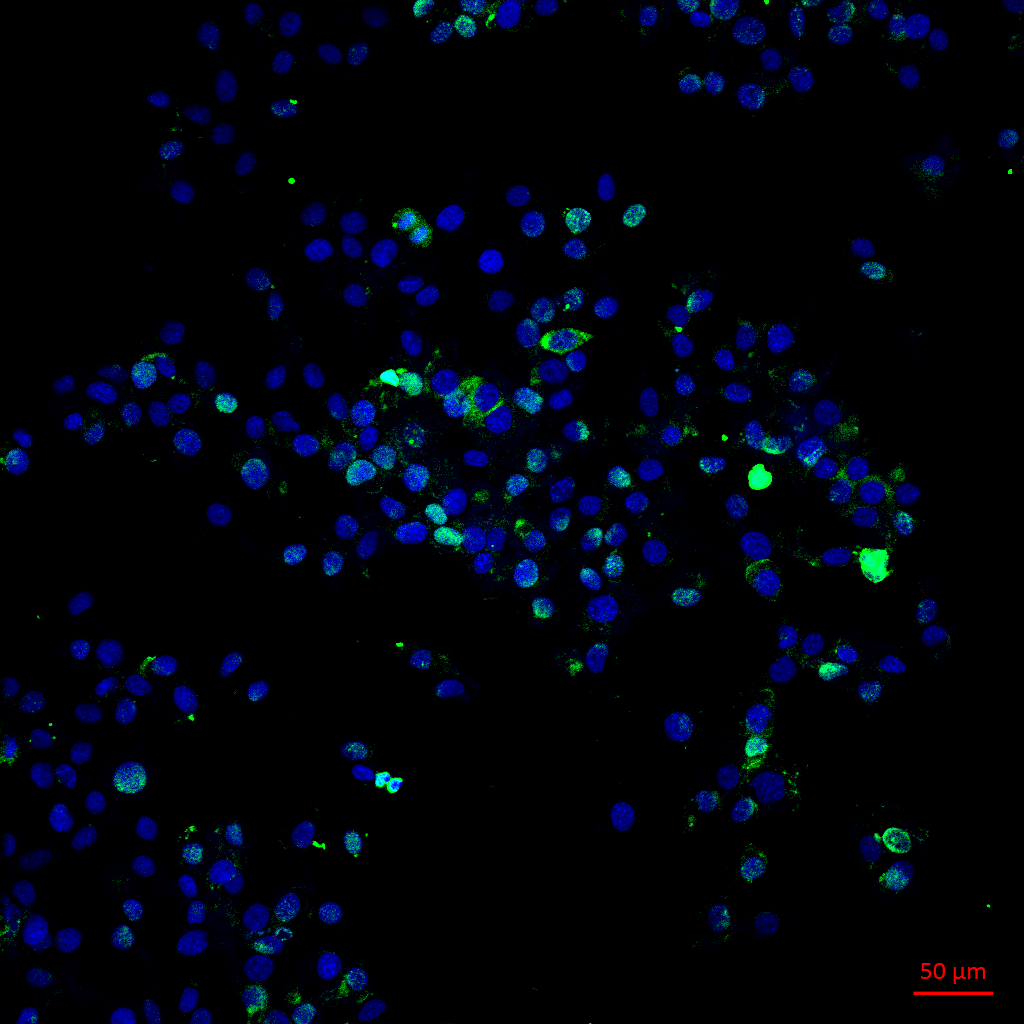

Supplement: Supplemental Information 2 [file peerj-11-15828-s002.zip › The raw data of immunofluorescence/figure-4c-si-1/Image 47│÷═╝si1_c1+2.tif]

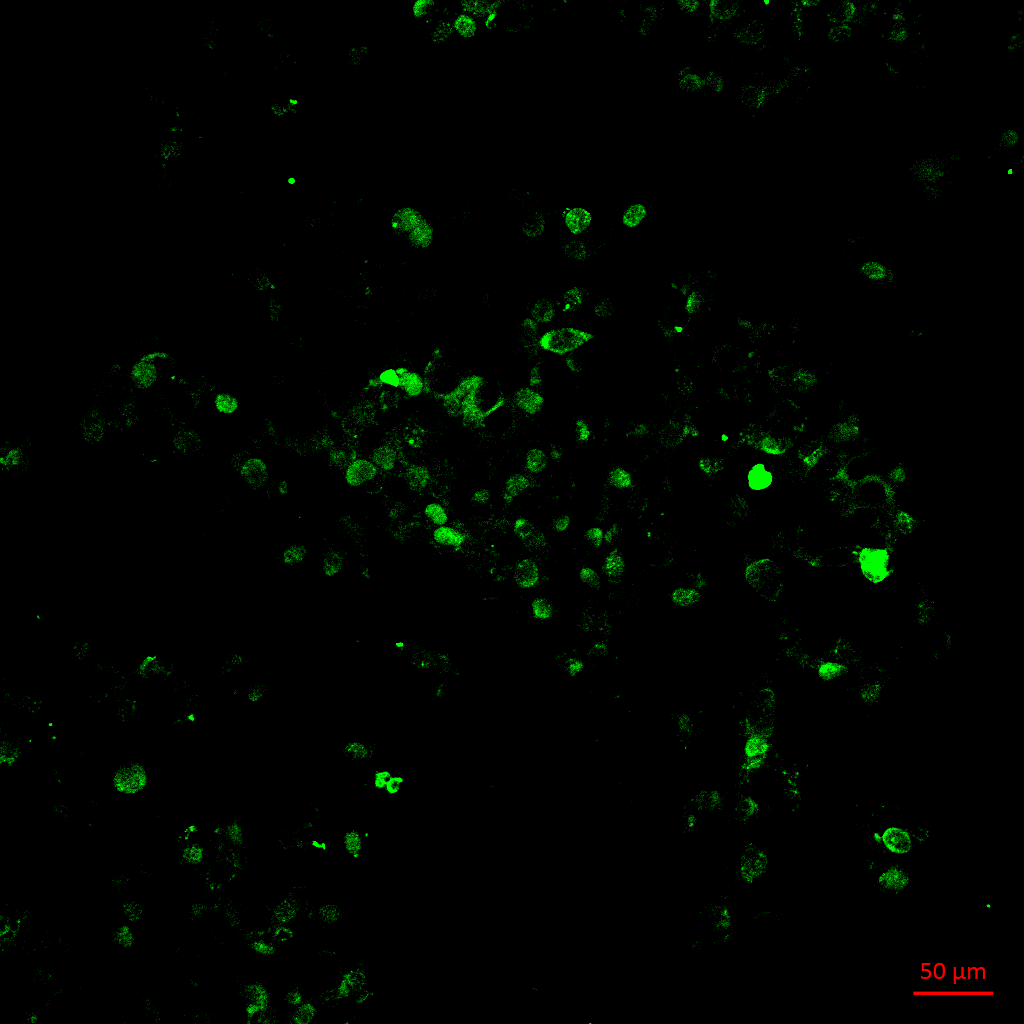

Supplement: Supplemental Information 2 [file peerj-11-15828-s002.zip › The raw data of immunofluorescence/figure-4c-si-1/Image 47│÷═╝si1_c1.tif]

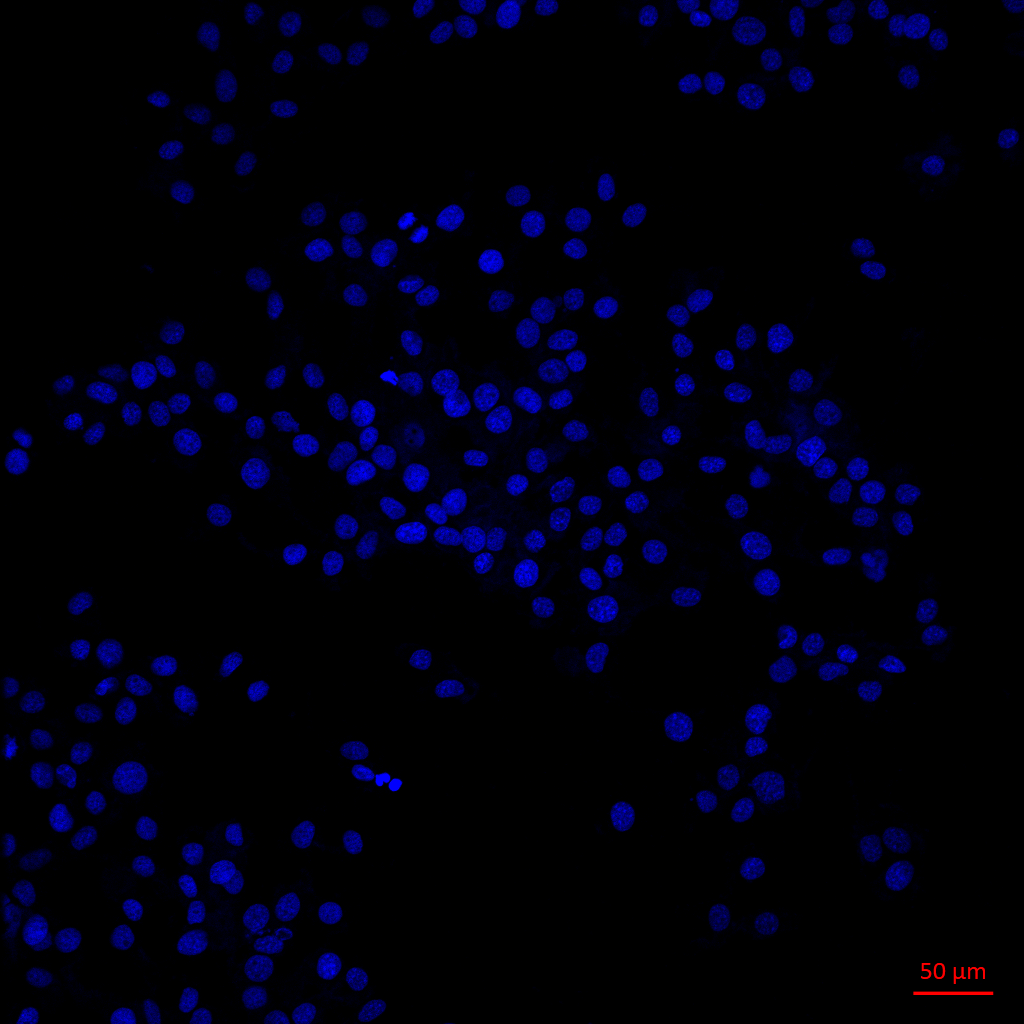

Supplement: Supplemental Information 2 [file peerj-11-15828-s002.zip › The raw data of immunofluorescence/figure-4c-si-1/Image 47│÷═╝si1_c2.tif]

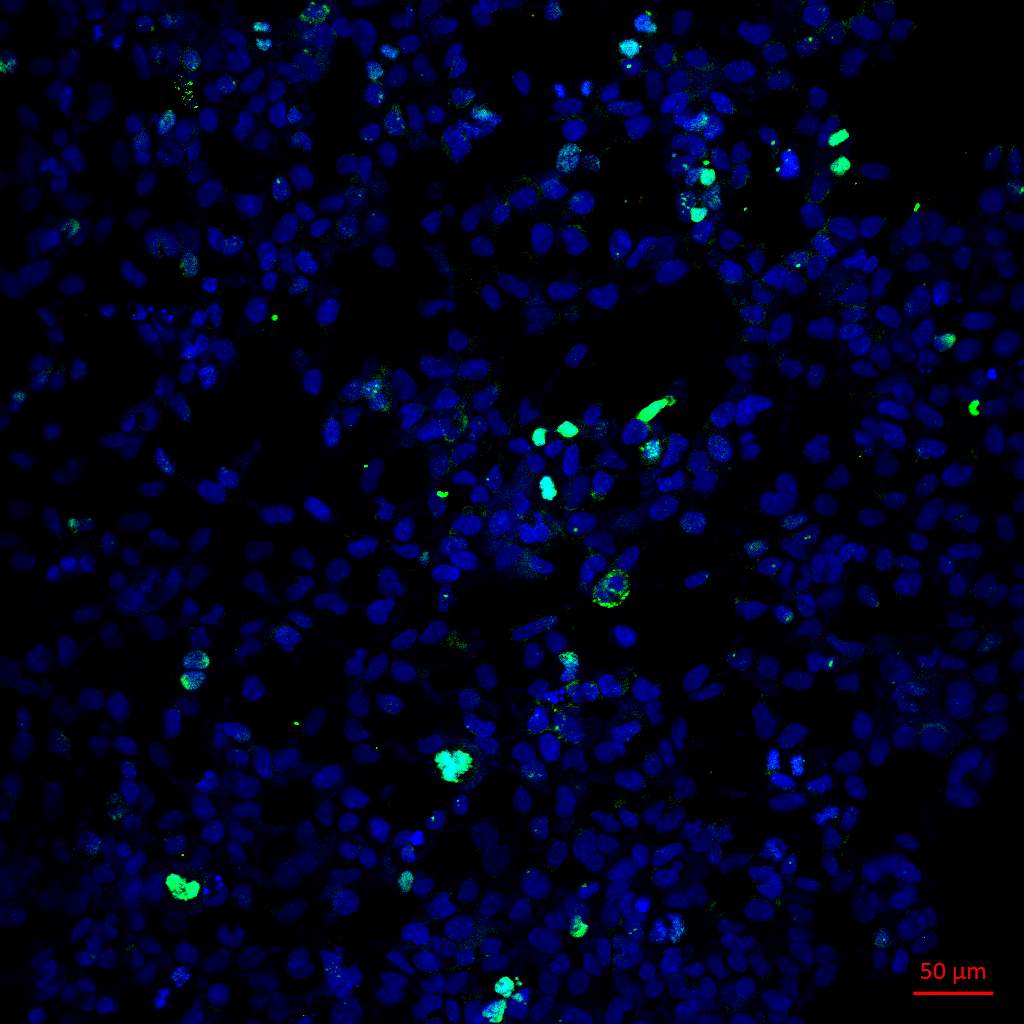

Supplement: Supplemental Information 2 [file peerj-11-15828-s002.zip › The raw data of immunofluorescence/figure-4c-si-2/Image 29│÷═╝si2_c1+2.tif]

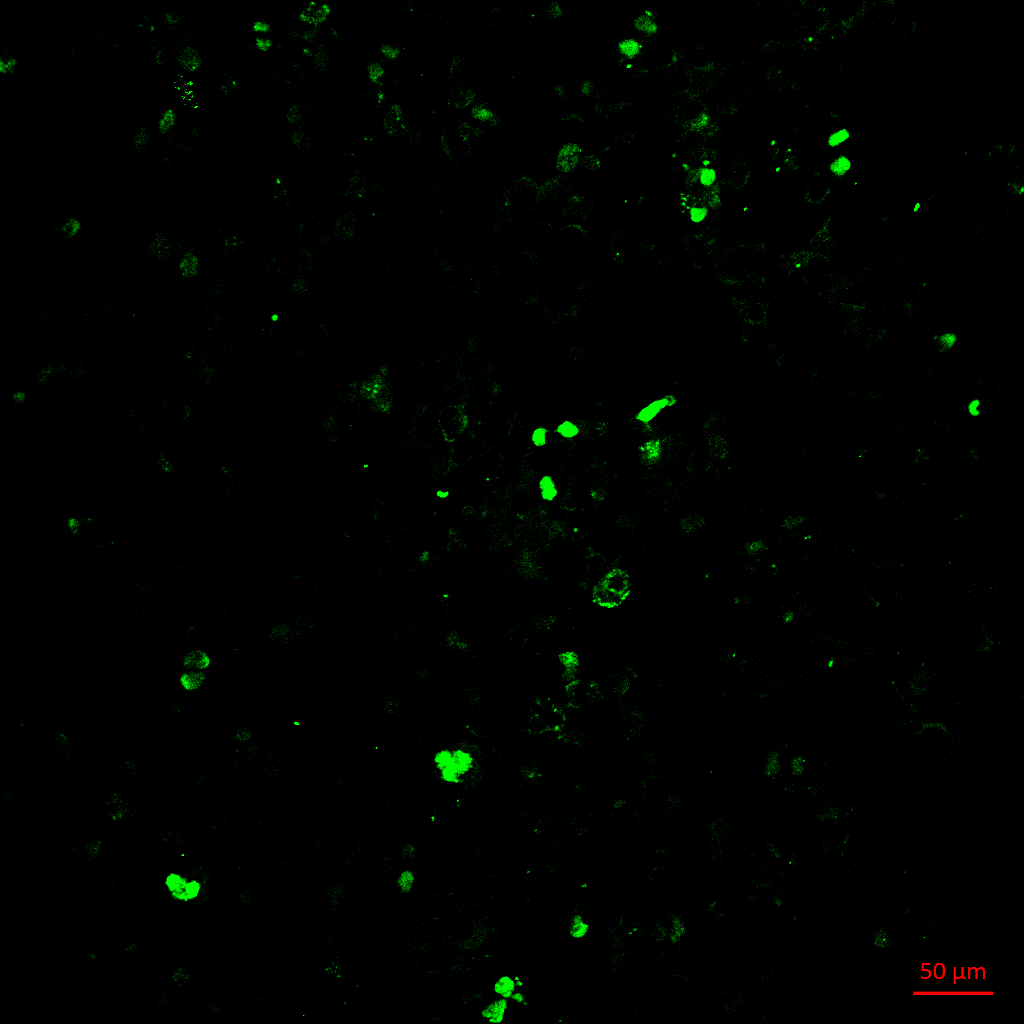

Supplement: Supplemental Information 2 [file peerj-11-15828-s002.zip › The raw data of immunofluorescence/figure-4c-si-2/Image 29│÷═╝si2_c1.tif]

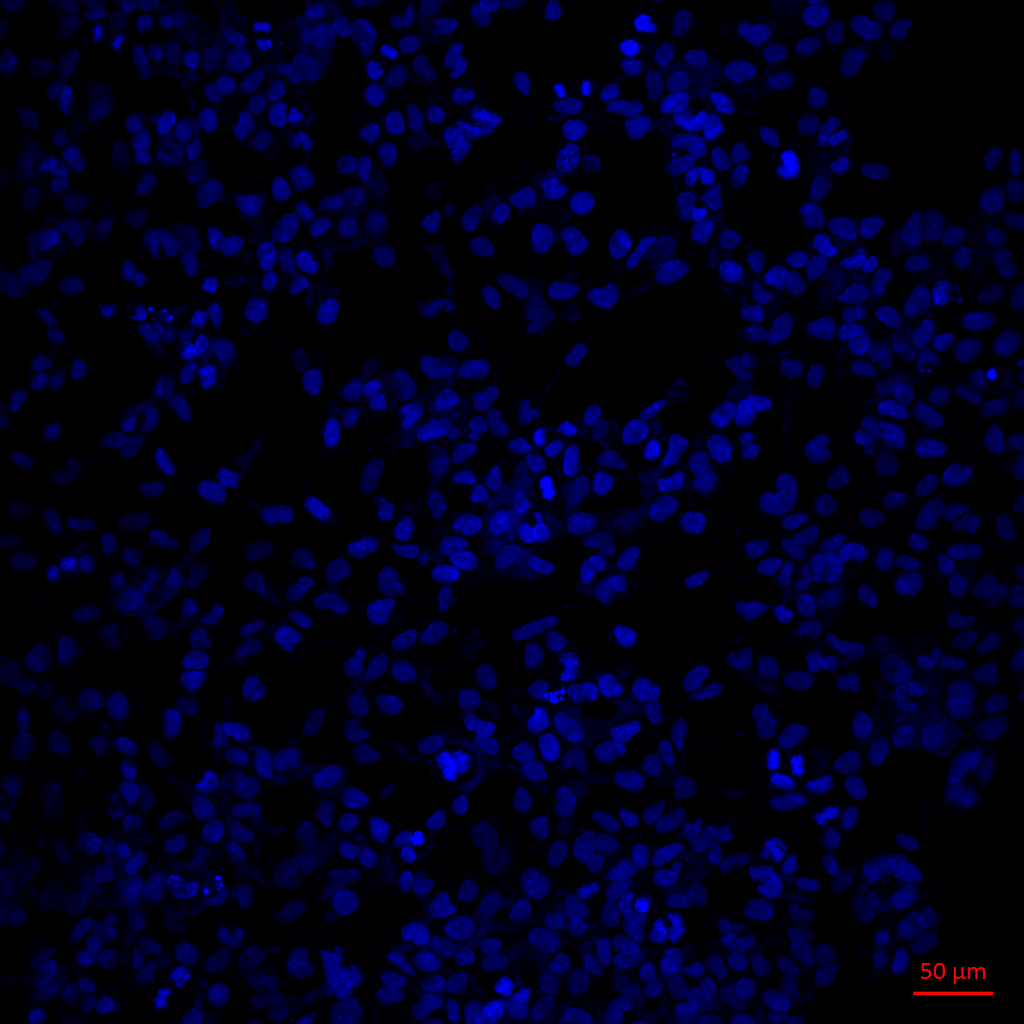

Supplement: Supplemental Information 2 [file peerj-11-15828-s002.zip › The raw data of immunofluorescence/figure-4c-si-2/Image 29│÷═╝si2_c2.tif]

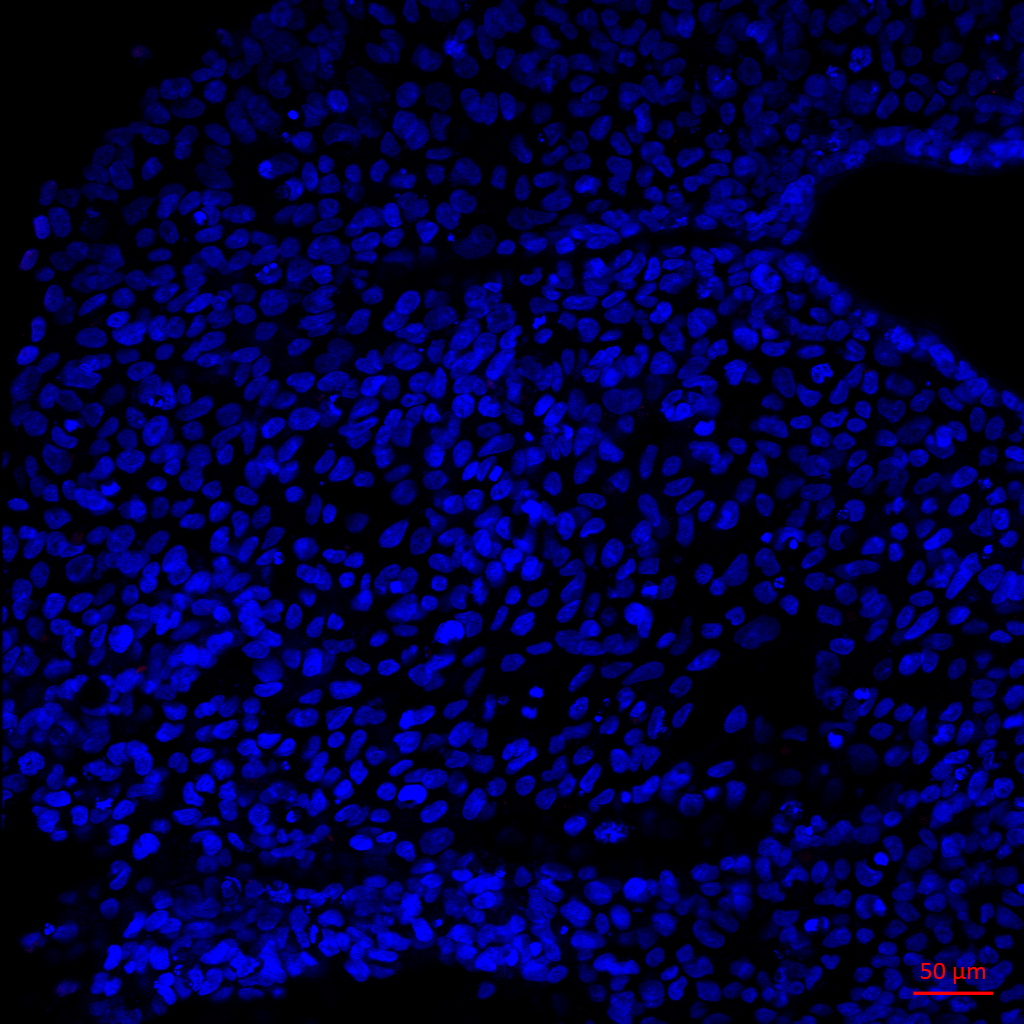

Supplement: Supplemental Information 2 [file peerj-11-15828-s002.zip › The raw data of immunofluorescence/figure1k-nc.tif]

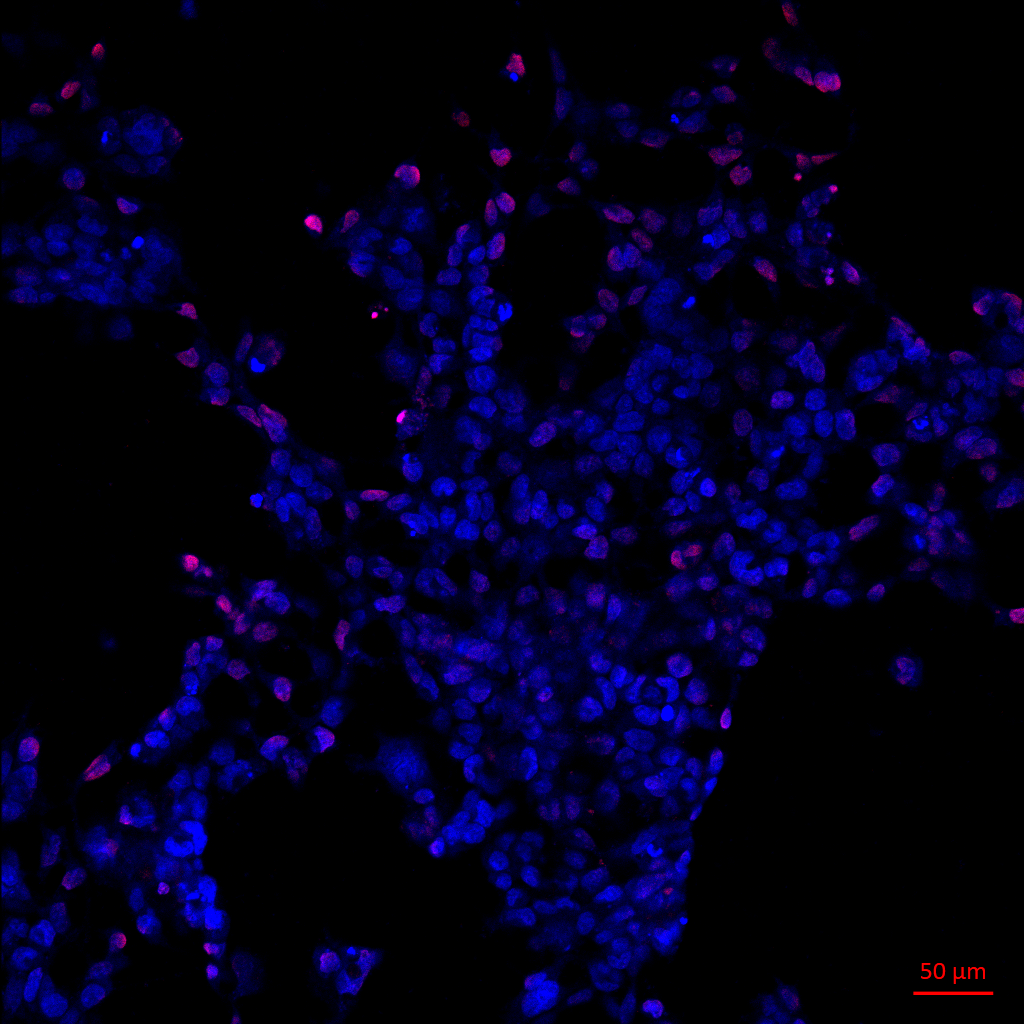

Supplement: Supplemental Information 2 [file peerj-11-15828-s002.zip › The raw data of immunofluorescence/figure1k-si-1.tif]

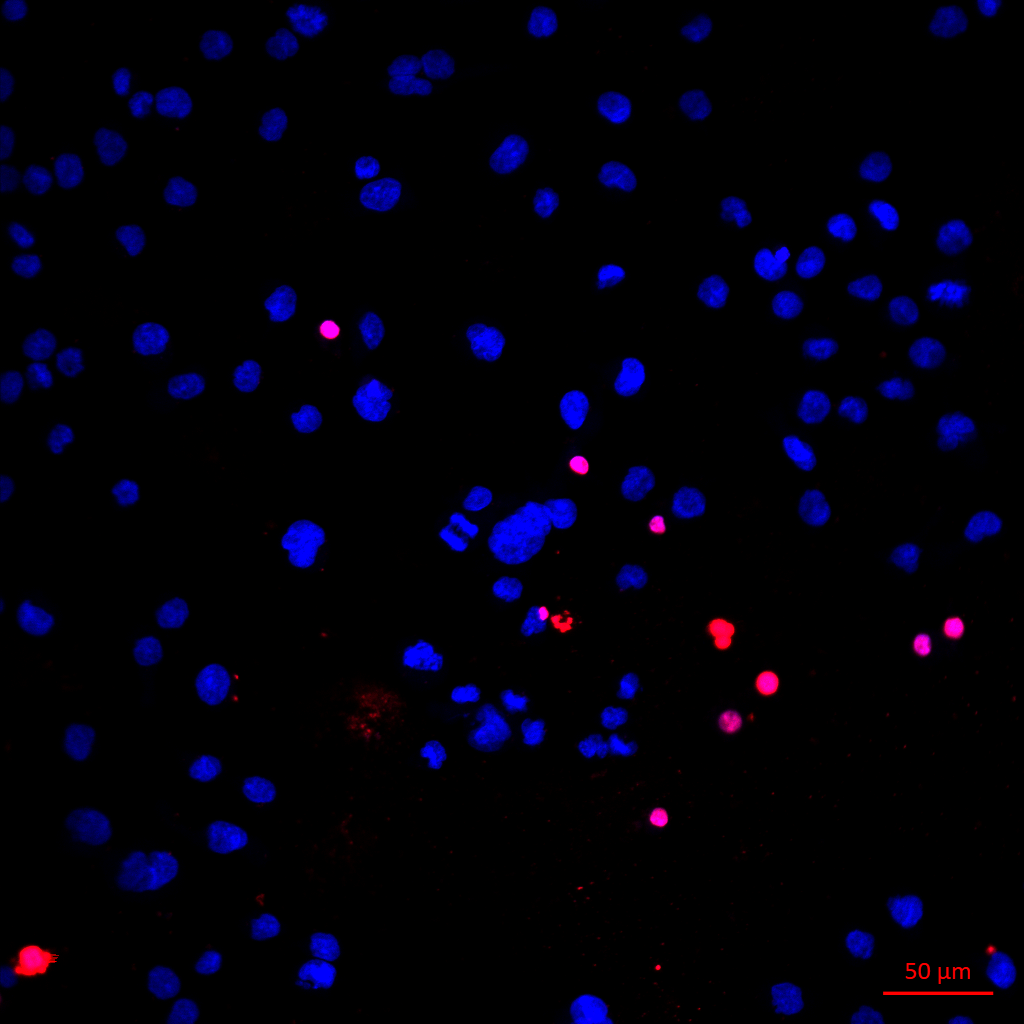

Supplement: Supplemental Information 2 [file peerj-11-15828-s002.zip › The raw data of immunofluorescence/figure1k-si-2.tif]

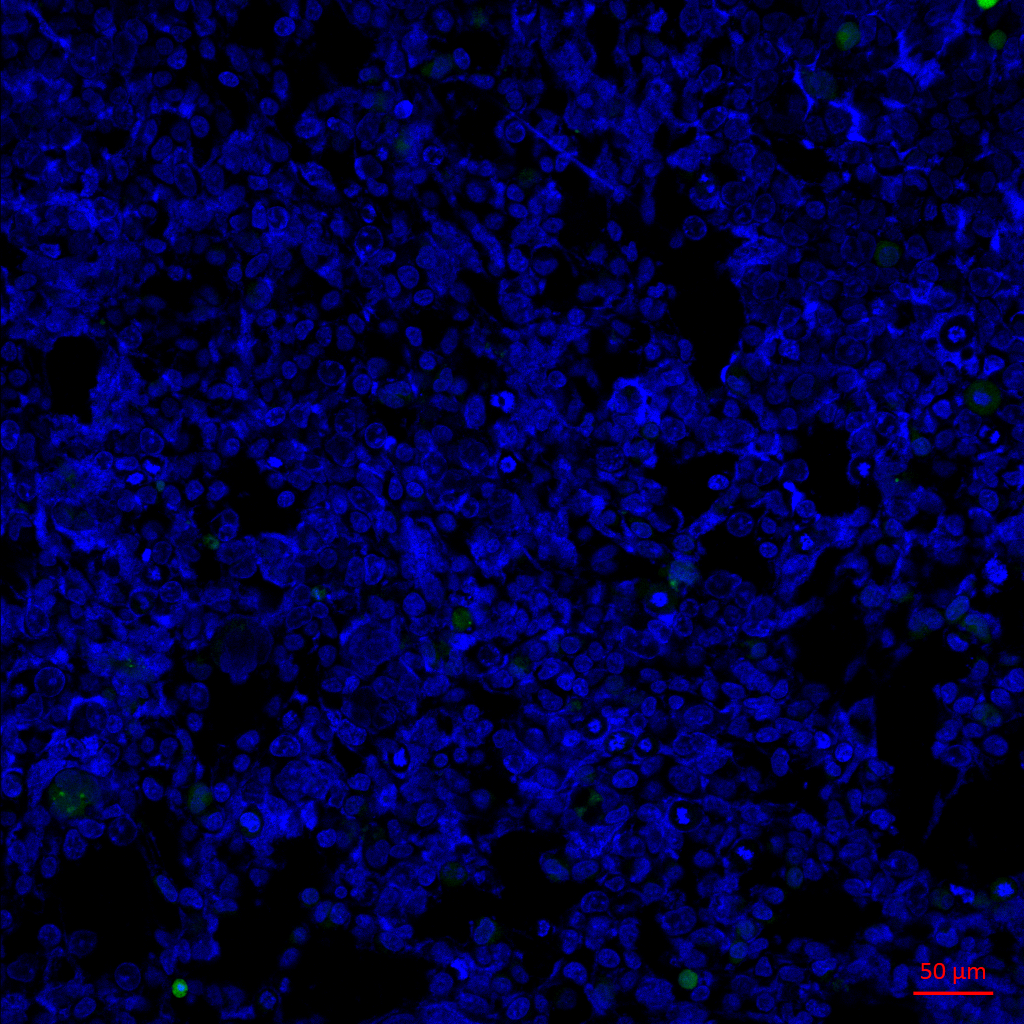

Supplement: Supplemental Information 2 [file peerj-11-15828-s002.zip › The raw data of immunofluorescence/figure4a-nc/Image 5│÷═╝nc_c1+2.tif]

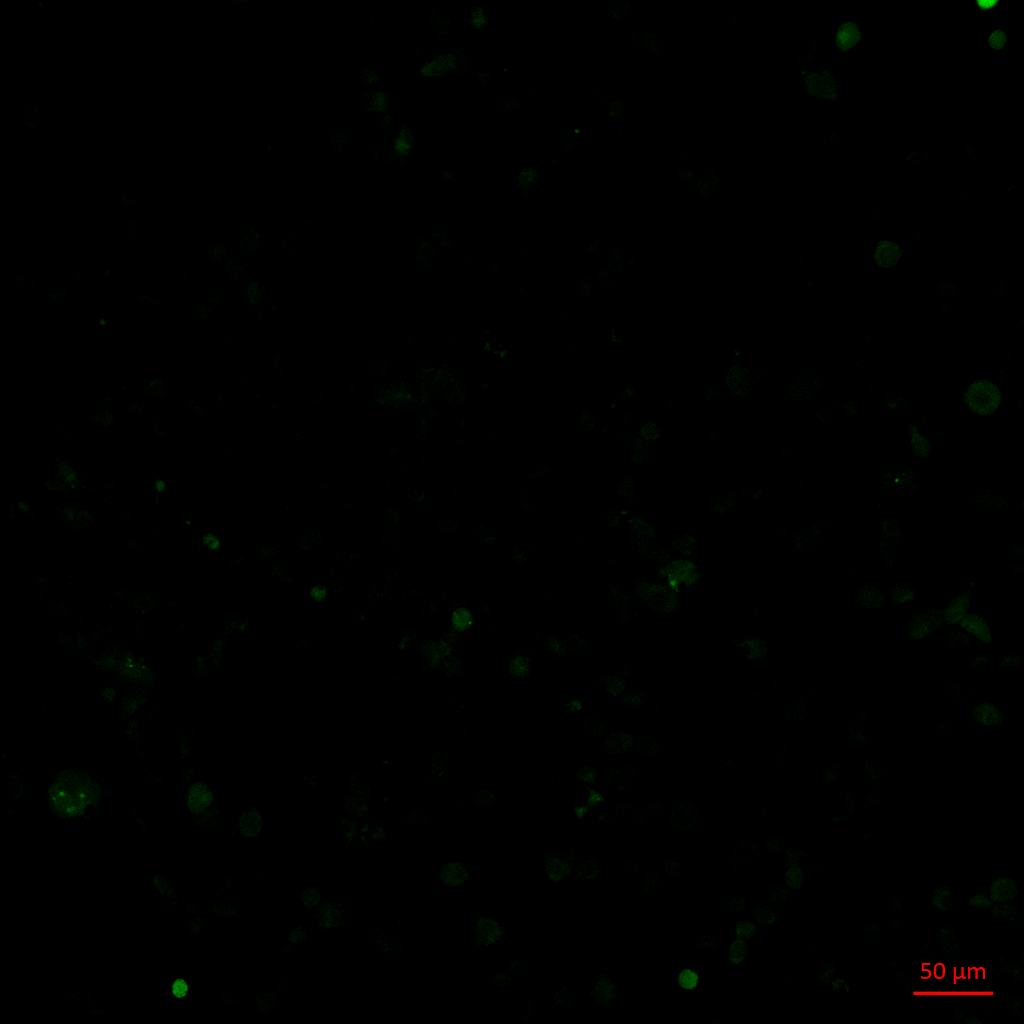

Supplement: Supplemental Information 2 [file peerj-11-15828-s002.zip › The raw data of immunofluorescence/figure4a-nc/Image 5│÷═╝nc_c1.tif]

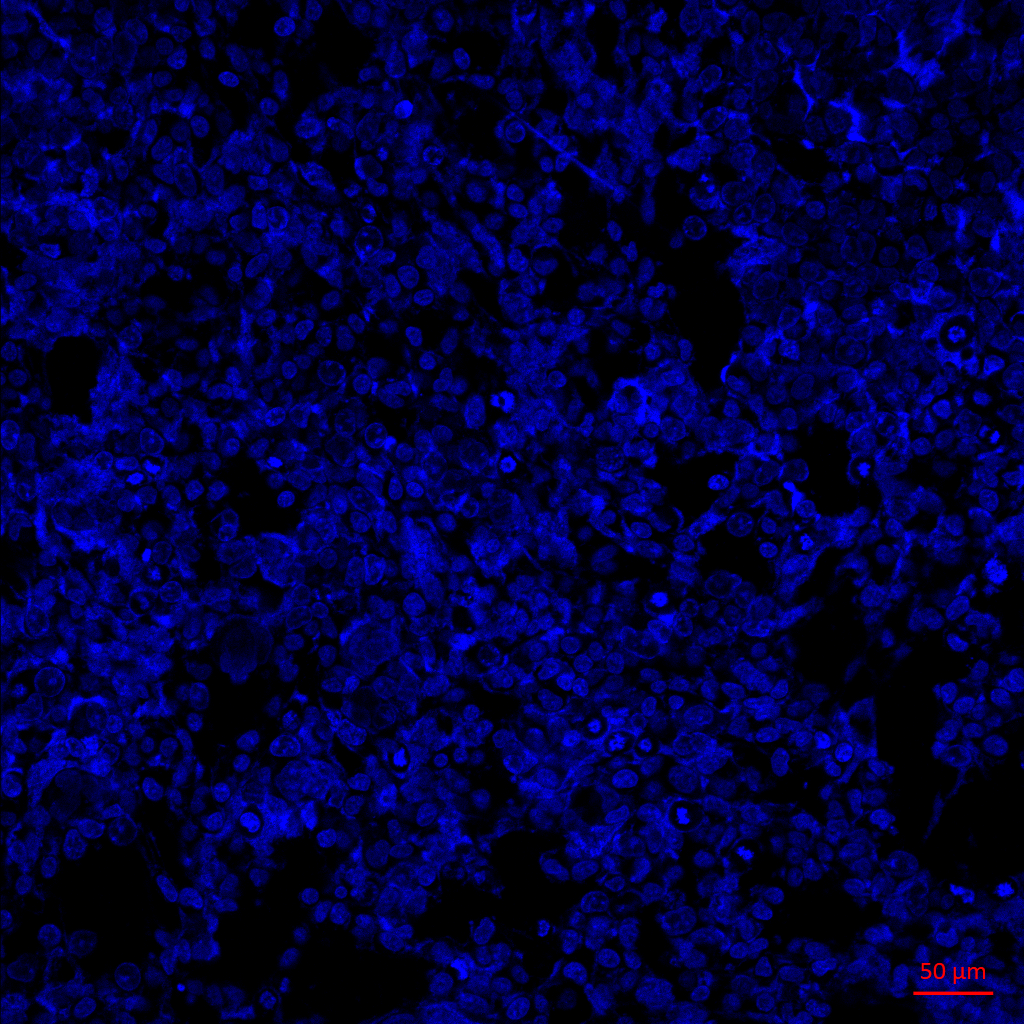

Supplement: Supplemental Information 2 [file peerj-11-15828-s002.zip › The raw data of immunofluorescence/figure4a-nc/Image 5│÷═╝nc_c2.tif]

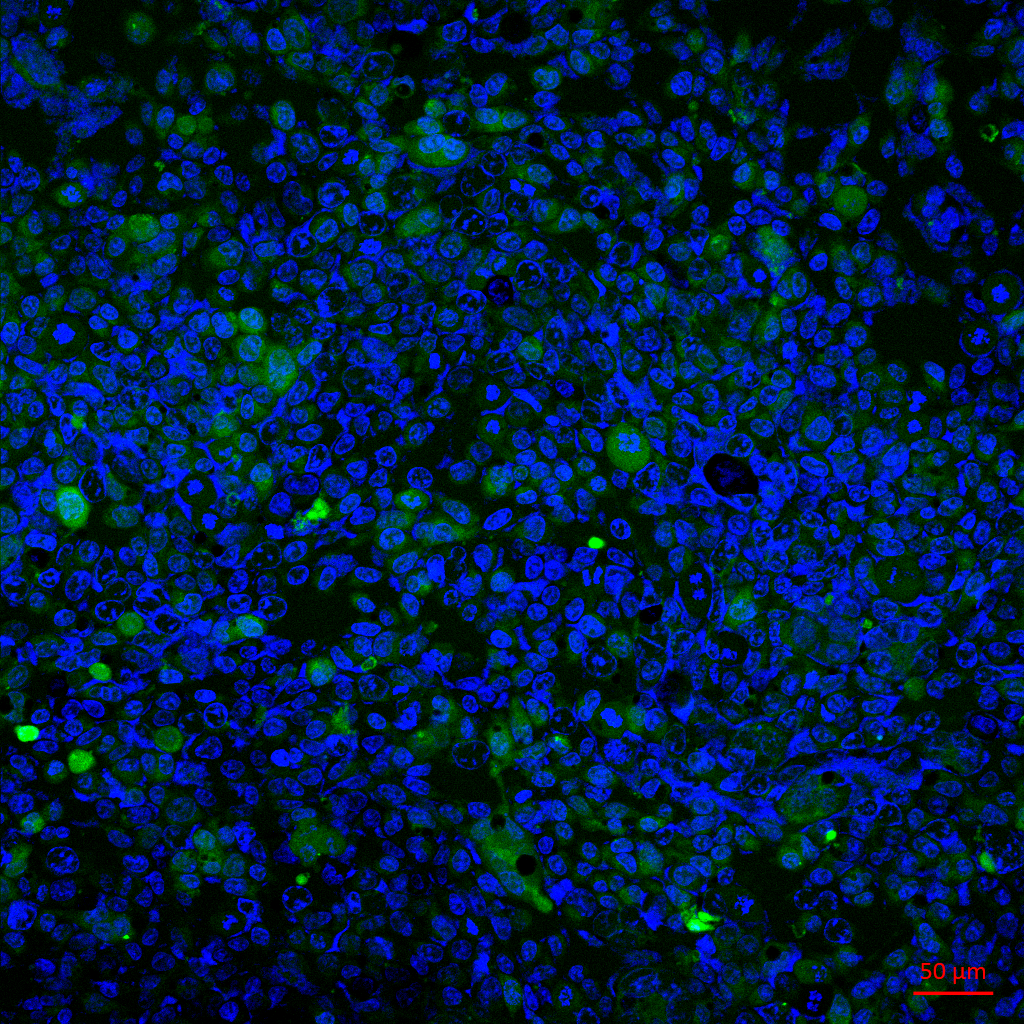

Supplement: Supplemental Information 2 [file peerj-11-15828-s002.zip › The raw data of immunofluorescence/figure4a-si-1/Image 14│÷═╝si-1_c1+2.tif]

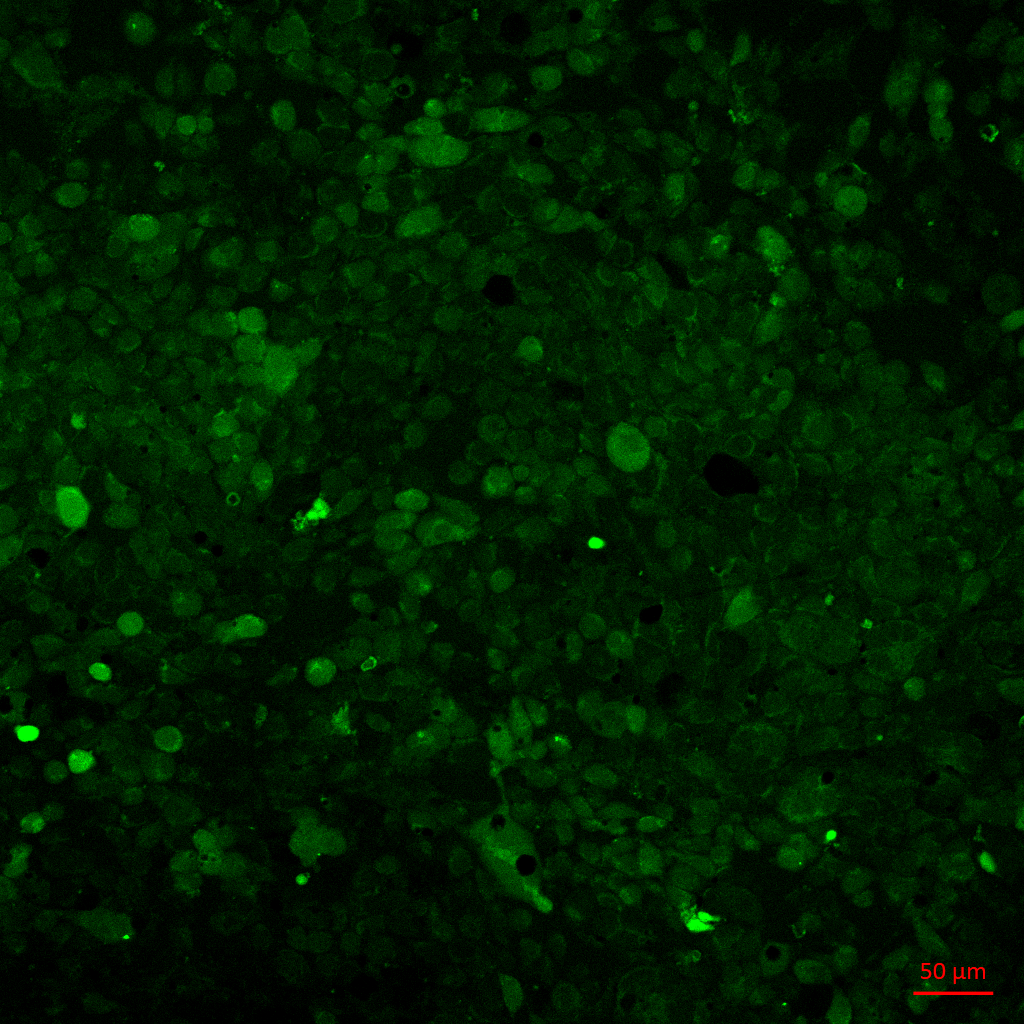

Supplement: Supplemental Information 2 [file peerj-11-15828-s002.zip › The raw data of immunofluorescence/figure4a-si-1/Image 14│÷═╝si-1_c1.tif]

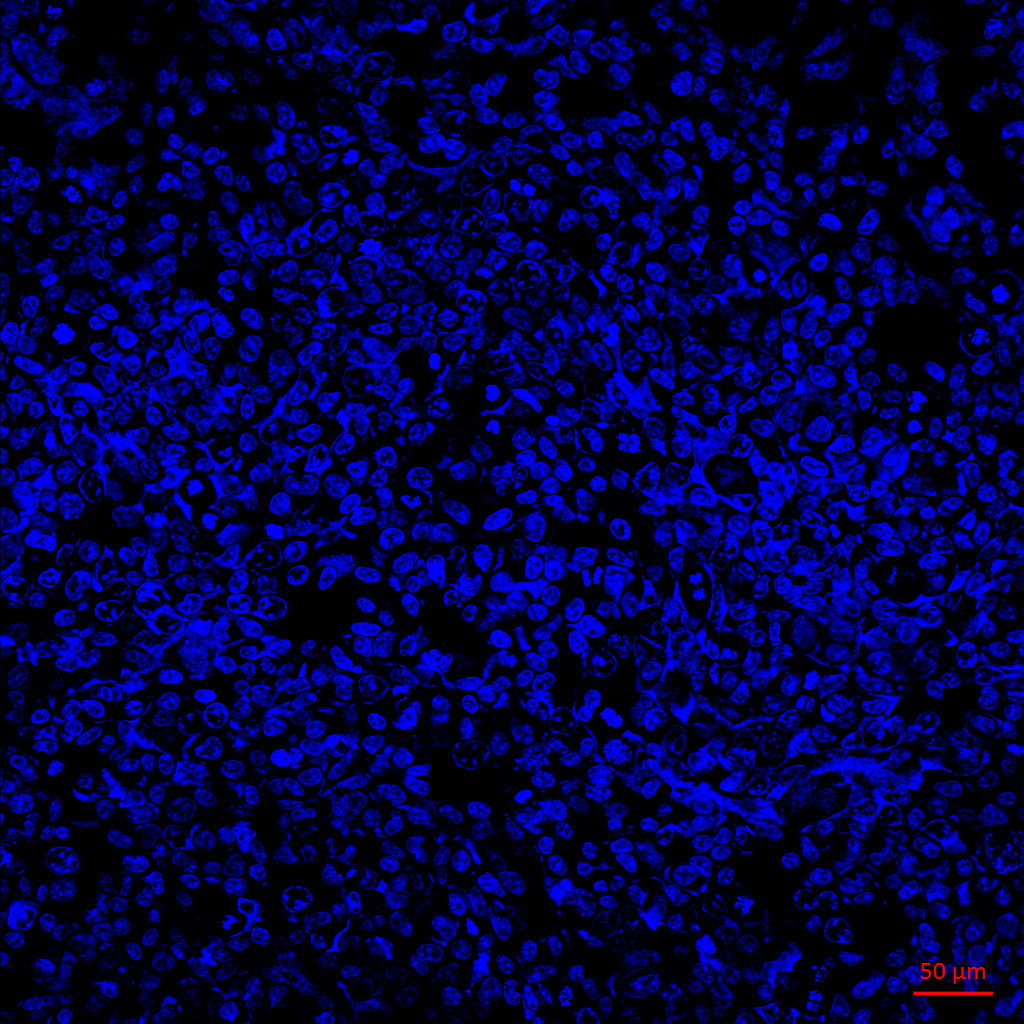

Supplement: Supplemental Information 2 [file peerj-11-15828-s002.zip › The raw data of immunofluorescence/figure4a-si-1/Image 14│÷═╝si-1_c2.tif]

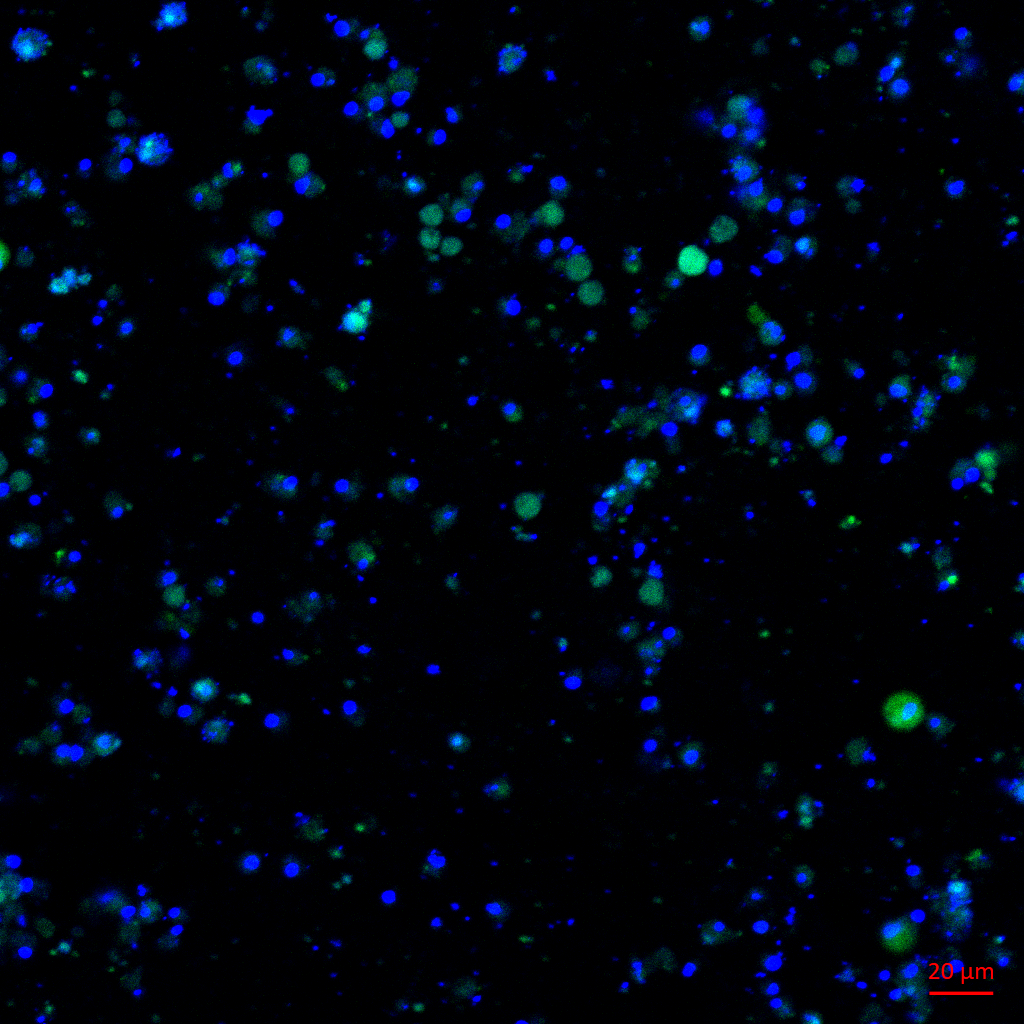

Supplement: Supplemental Information 2 [file peerj-11-15828-s002.zip › The raw data of immunofluorescence/figure4a-si-2/Image 47│÷═╝si-2_c1+2.tif]

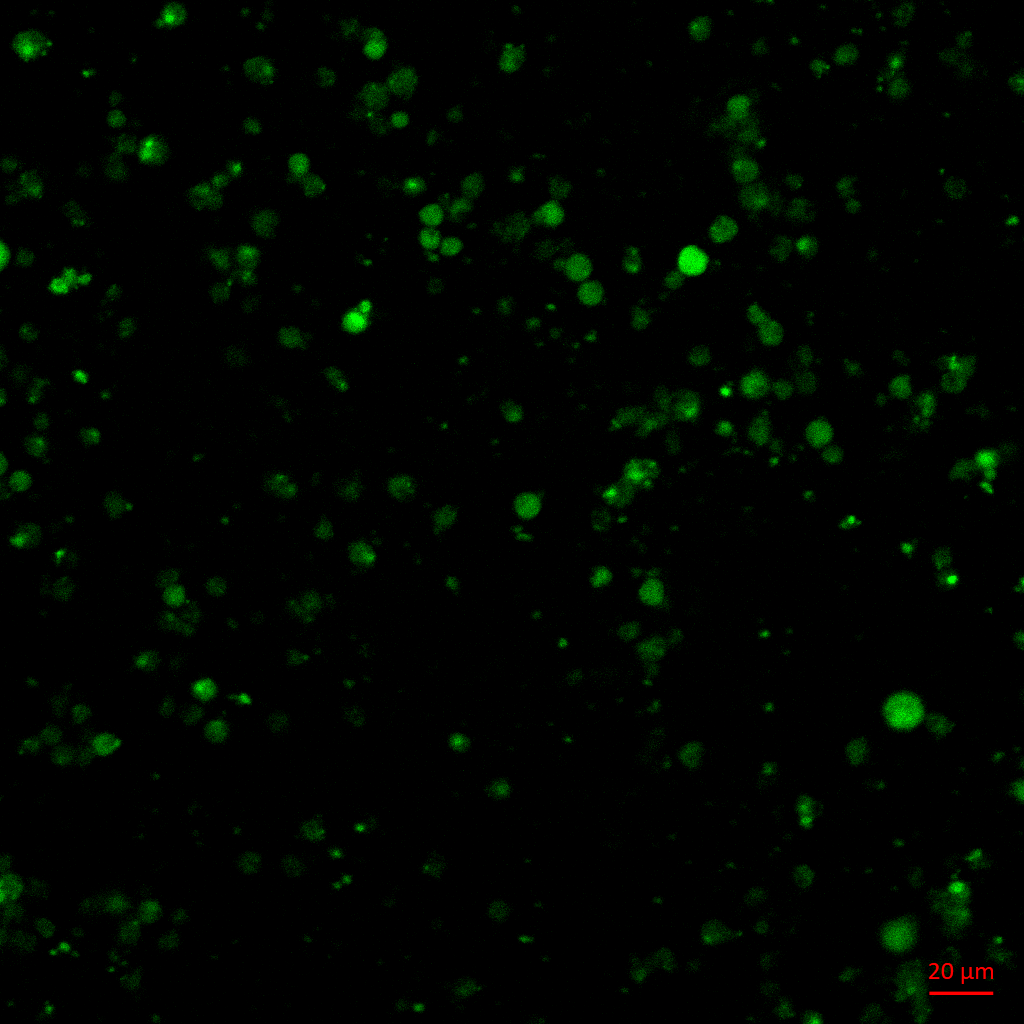

Supplement: Supplemental Information 2 [file peerj-11-15828-s002.zip › The raw data of immunofluorescence/figure4a-si-2/Image 47│÷═╝si-2_c1.tif]

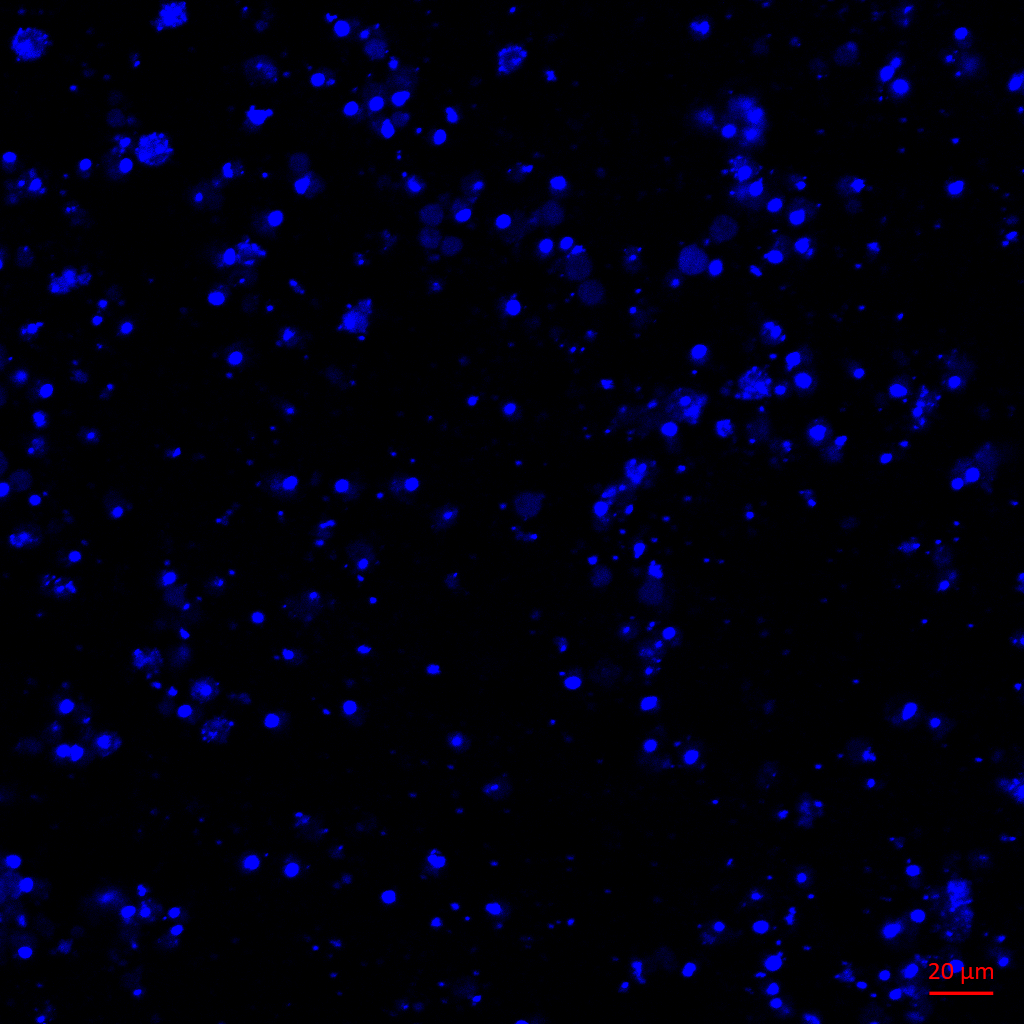

Supplement: Supplemental Information 2 [file peerj-11-15828-s002.zip › The raw data of immunofluorescence/figure4a-si-2/Image 47│÷═╝si-2_c2.tif]

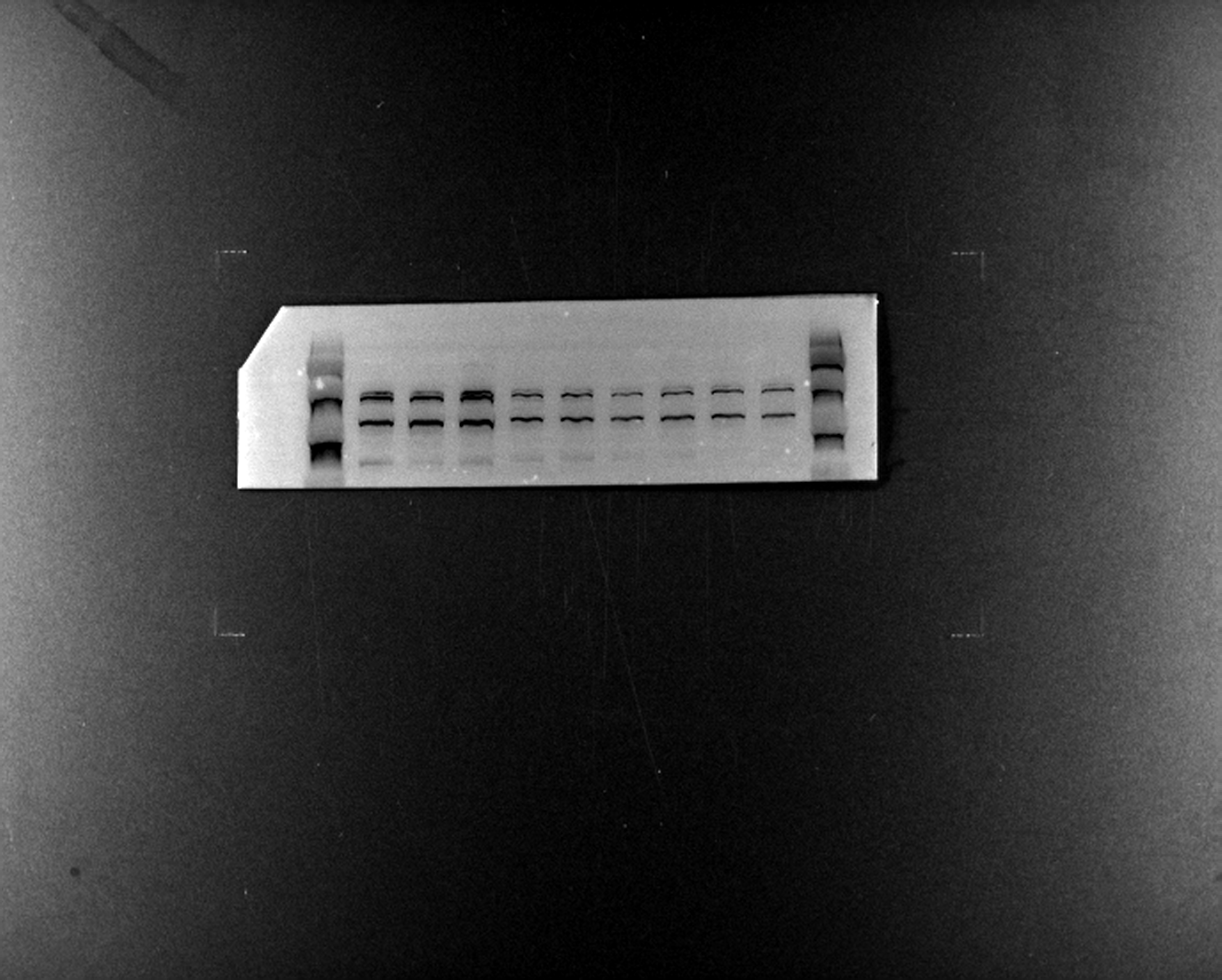

Supplement: Supplemental Information 4 [file peerj-11-15828-s004.zip › The raw data of Western blot/fig1b-bag3.tif]

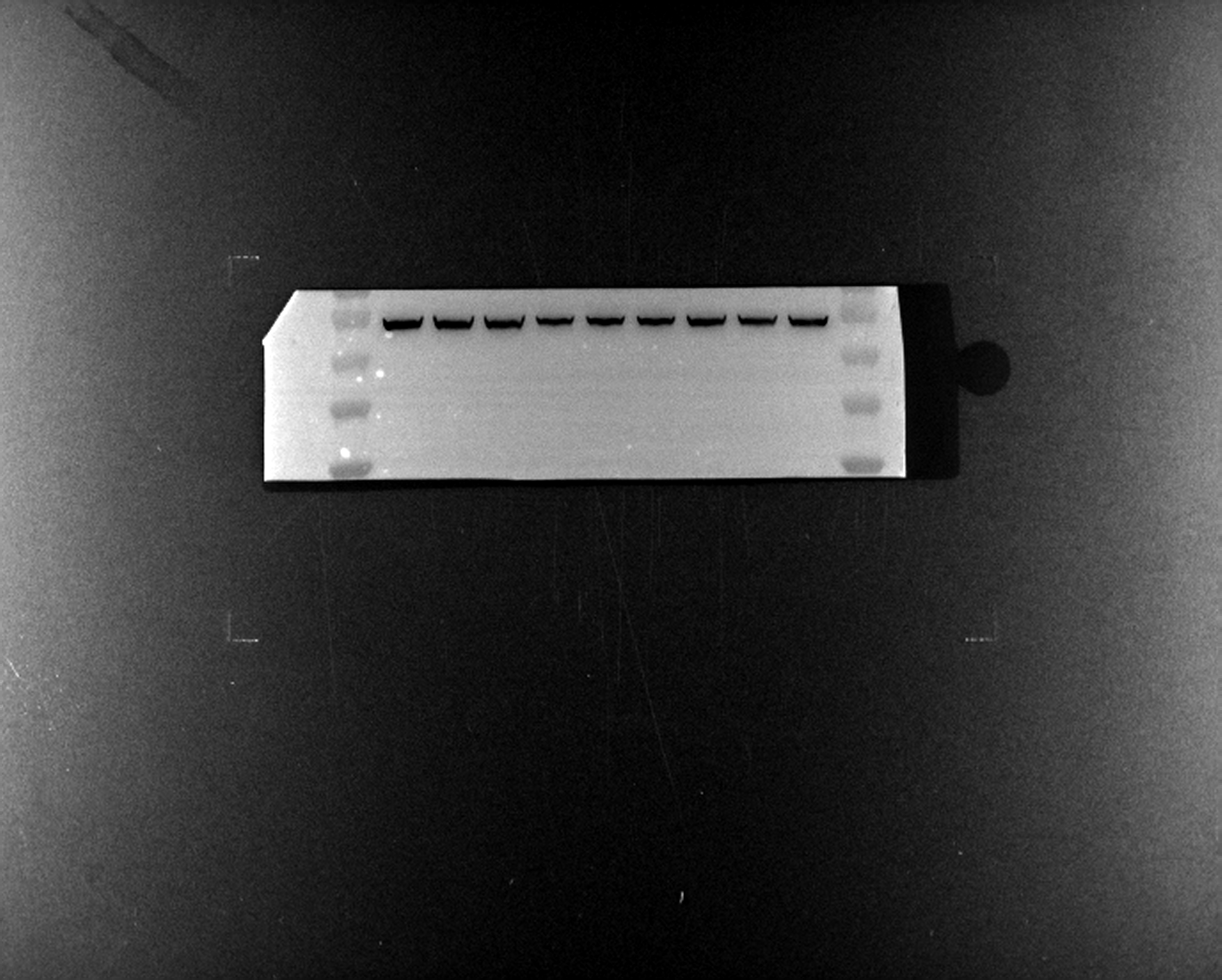

Supplement: Supplemental Information 4 [file peerj-11-15828-s004.zip › The raw data of Western blot/fig1b-tubulin.tif]

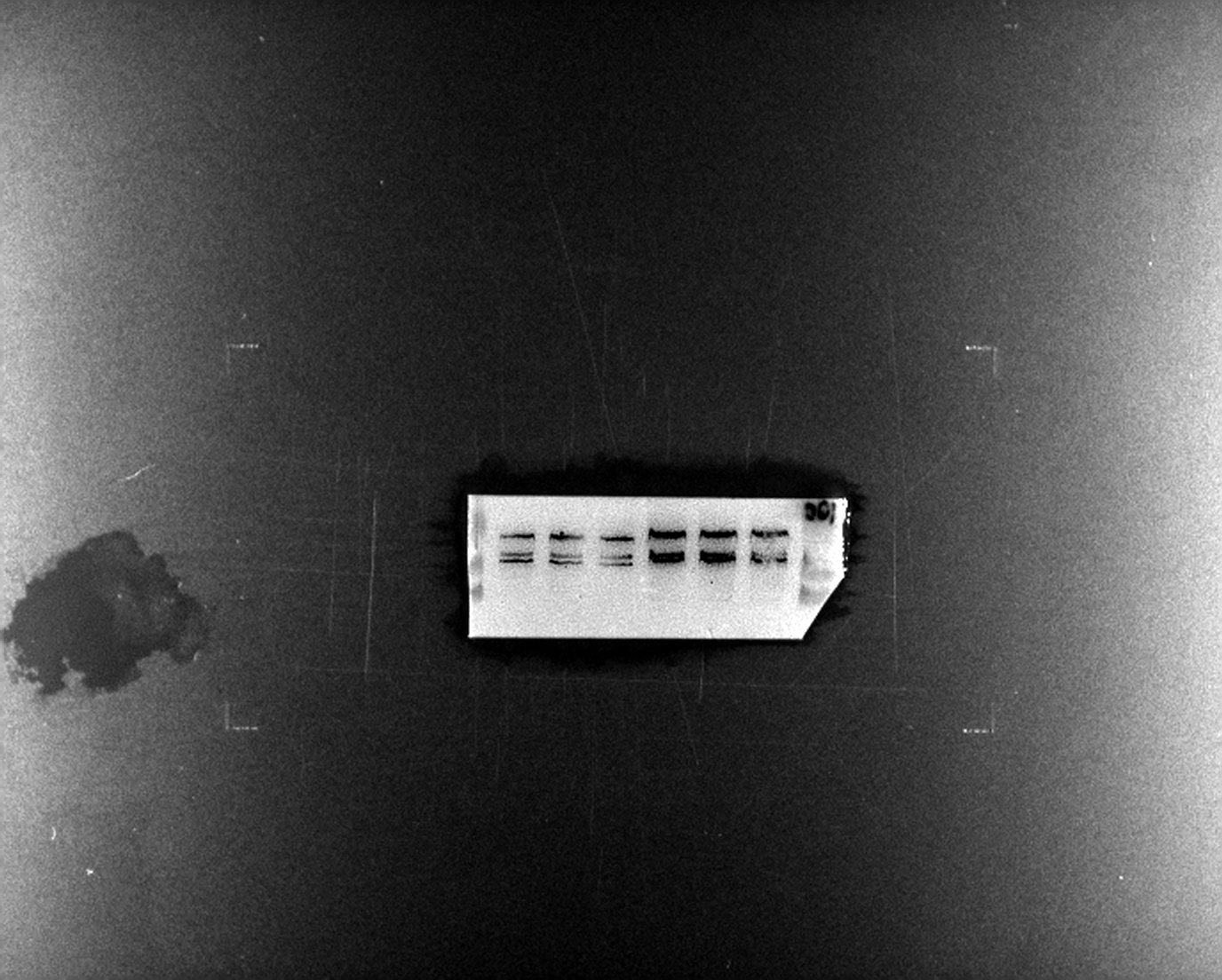

Supplement: Supplemental Information 4 [file peerj-11-15828-s004.zip › The raw data of Western blot/fig2a-bag3.tif]

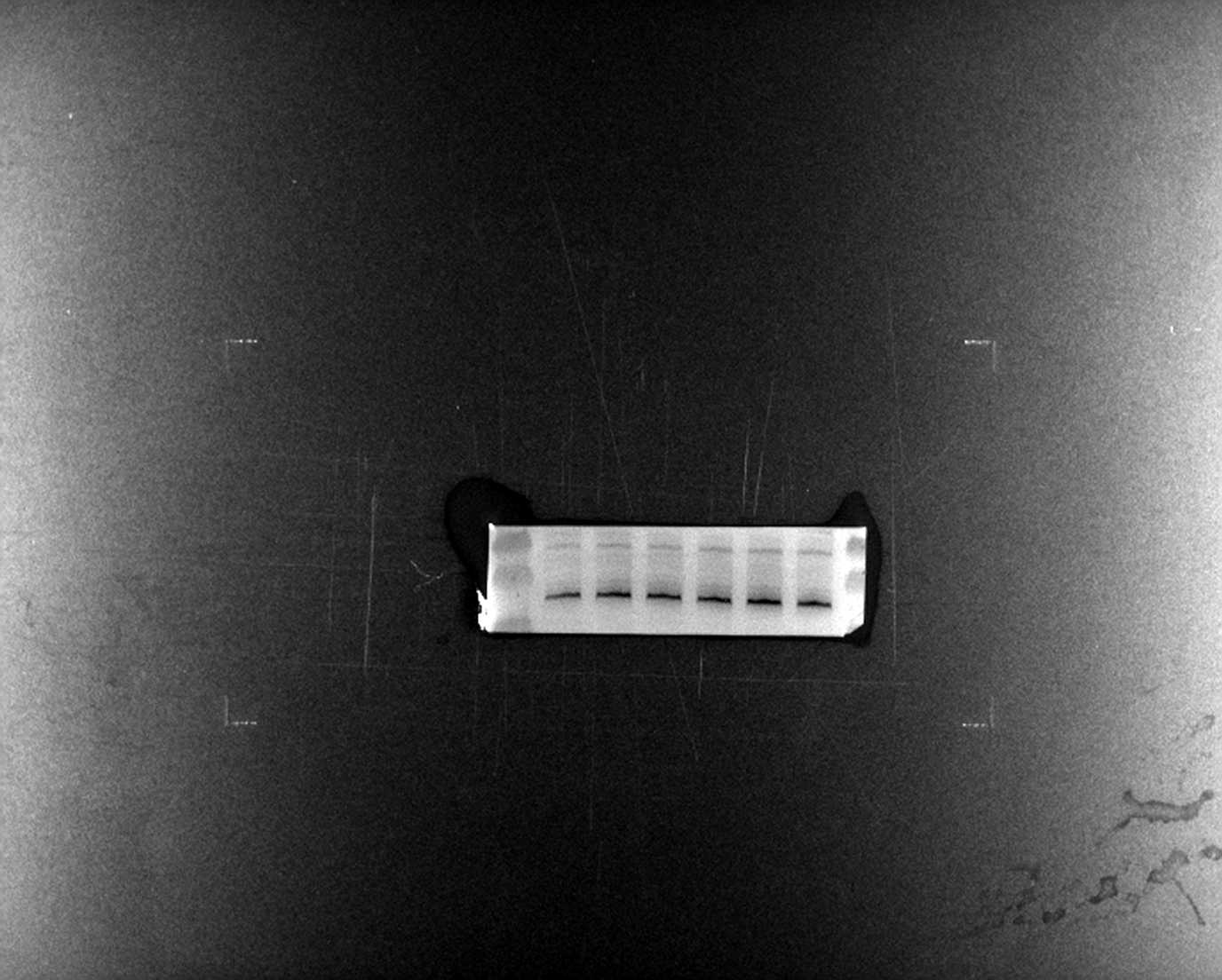

Supplement: Supplemental Information 4 [file peerj-11-15828-s004.zip › The raw data of Western blot/fig2a-tubulin.tif]

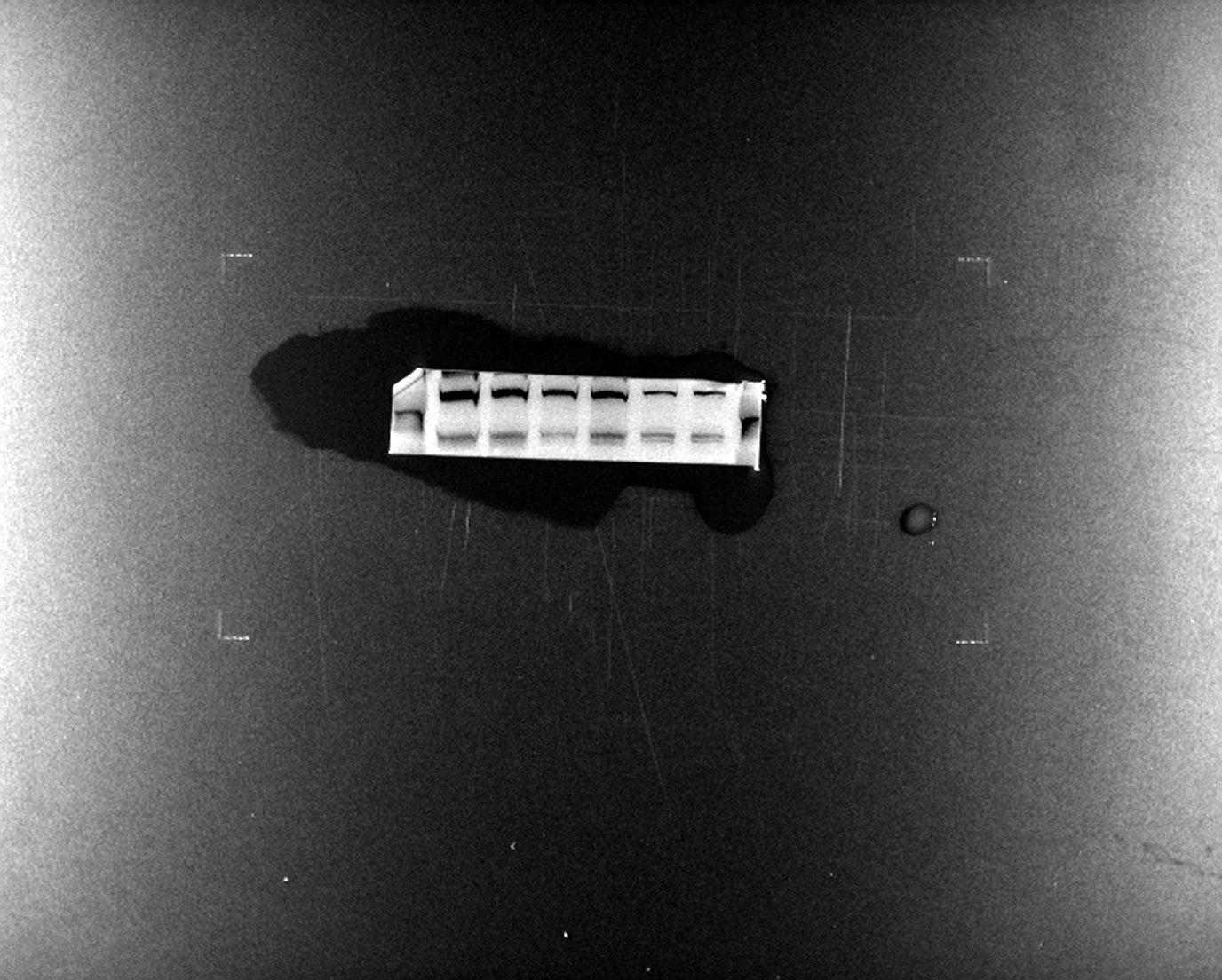

Supplement: Supplemental Information 4 [file peerj-11-15828-s004.zip › The raw data of Western blot/fig3d-ints7.tif]

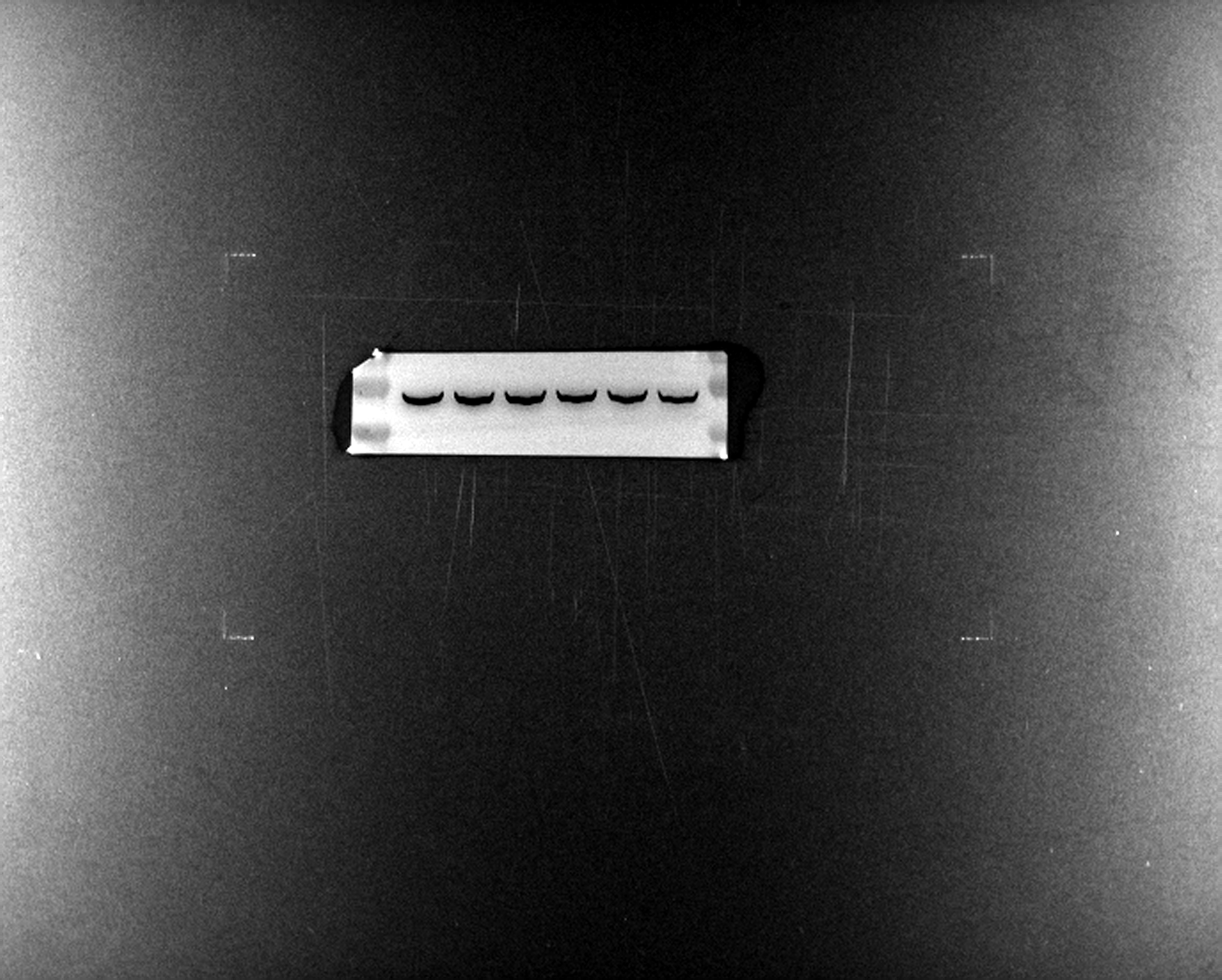

Supplement: Supplemental Information 4 [file peerj-11-15828-s004.zip › The raw data of Western blot/fig3d-tubulin.tif]

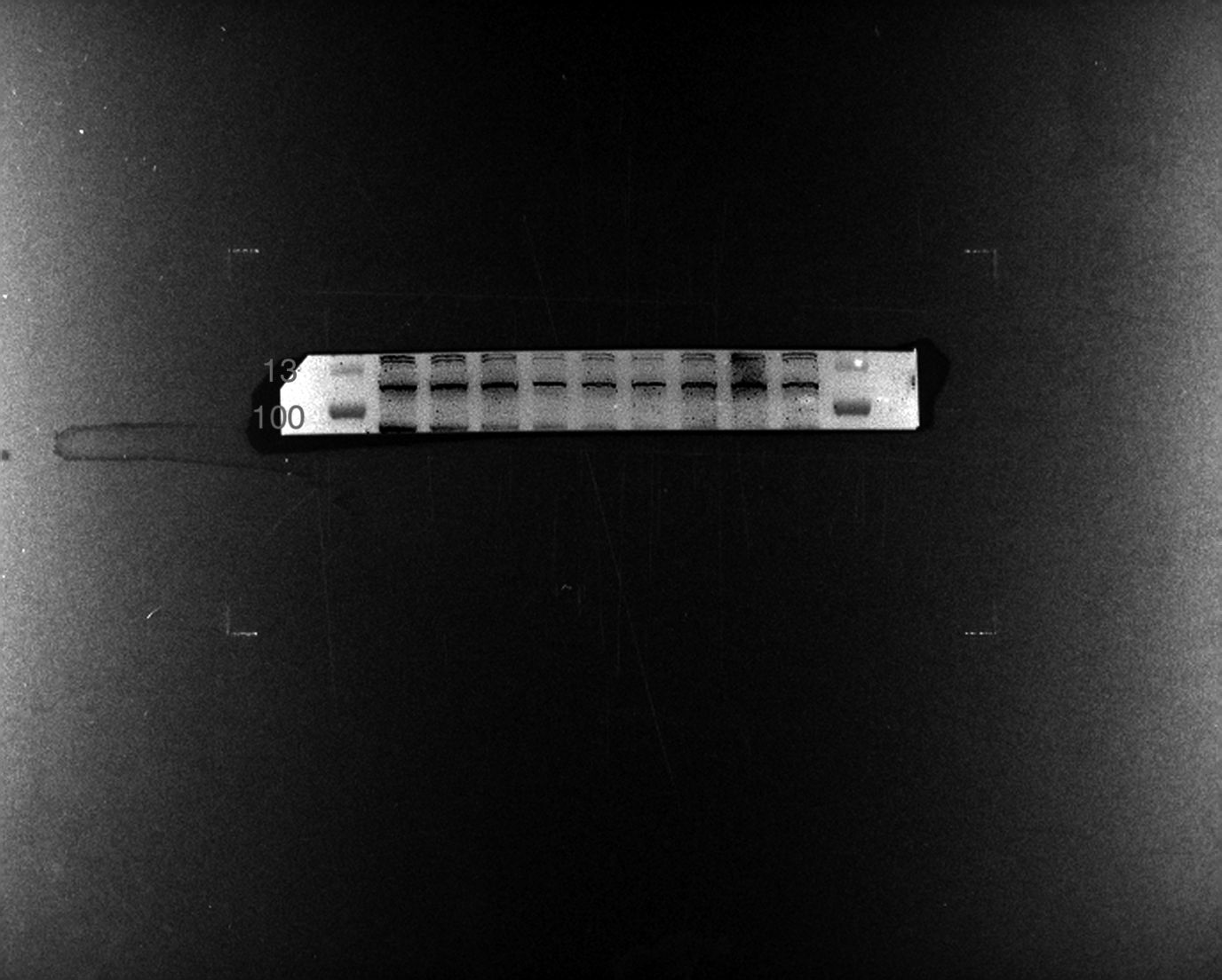

Supplement: Supplemental Information 4 [file peerj-11-15828-s004.zip › The raw data of Western blot/fig3f-ints7.tif]

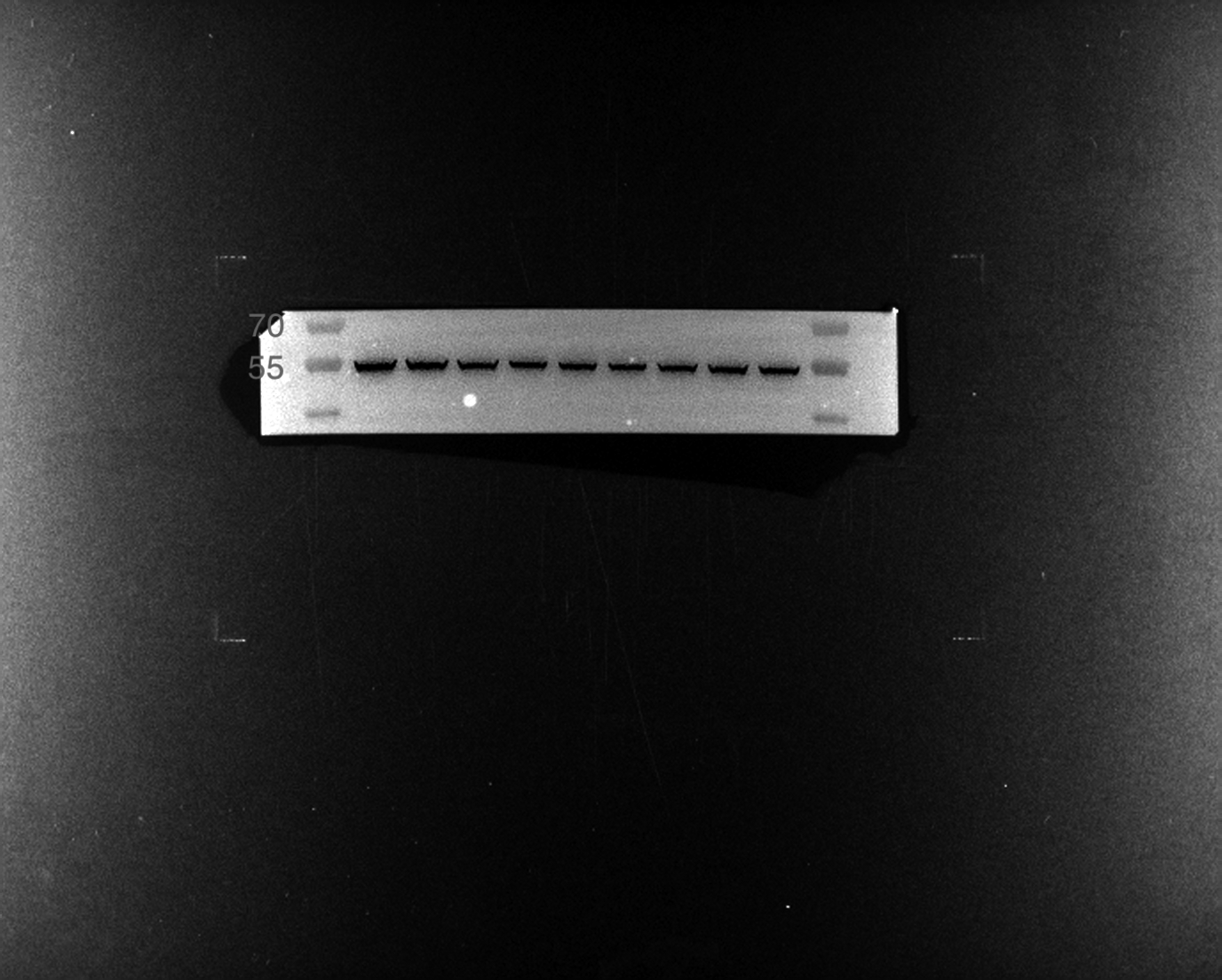

Supplement: Supplemental Information 4 [file peerj-11-15828-s004.zip › The raw data of Western blot/fig3f-tubulin.tif]

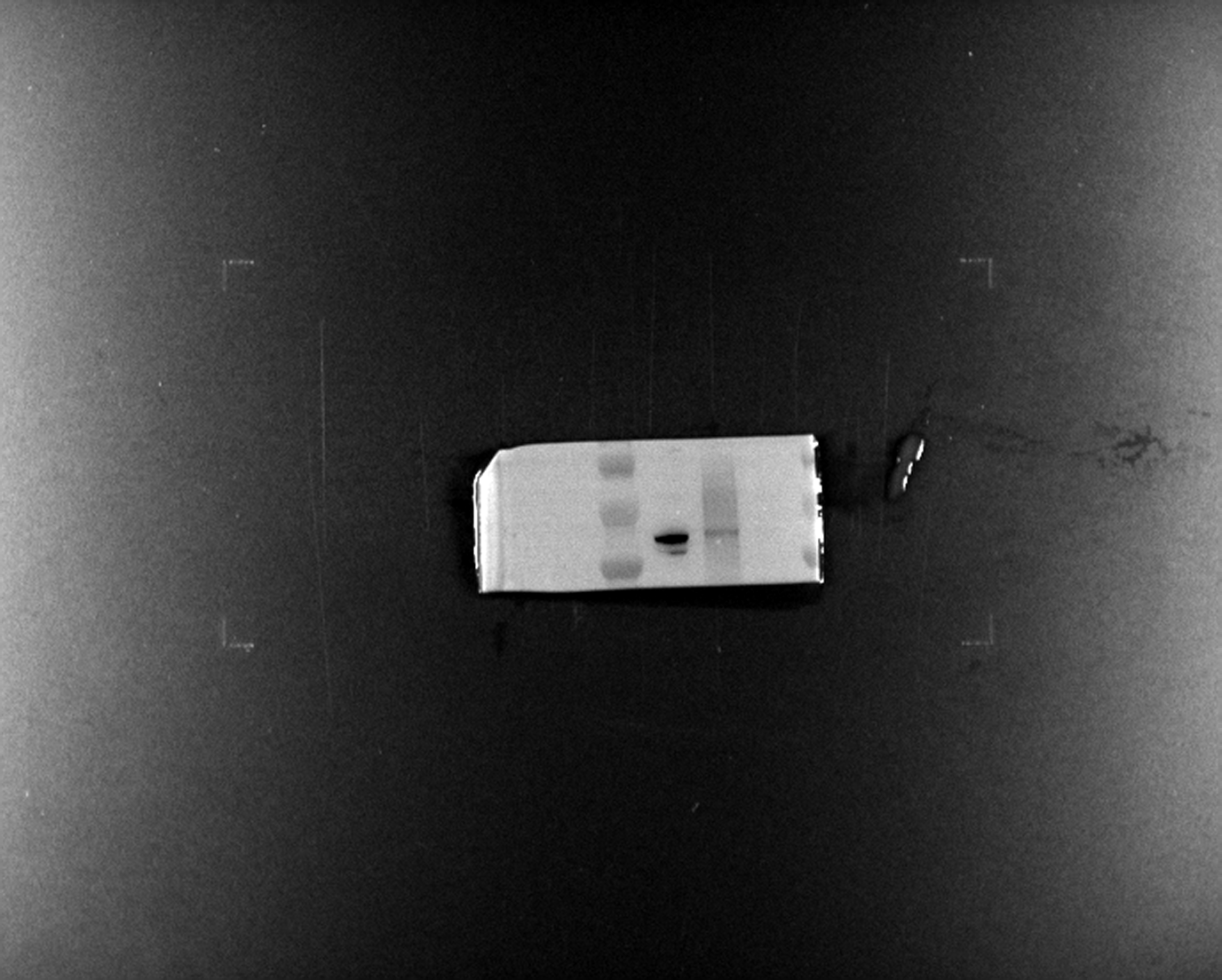

Supplement: Supplemental Information 4 [file peerj-11-15828-s004.zip › The raw data of Western blot/figure3c-bag3-down.Tif]

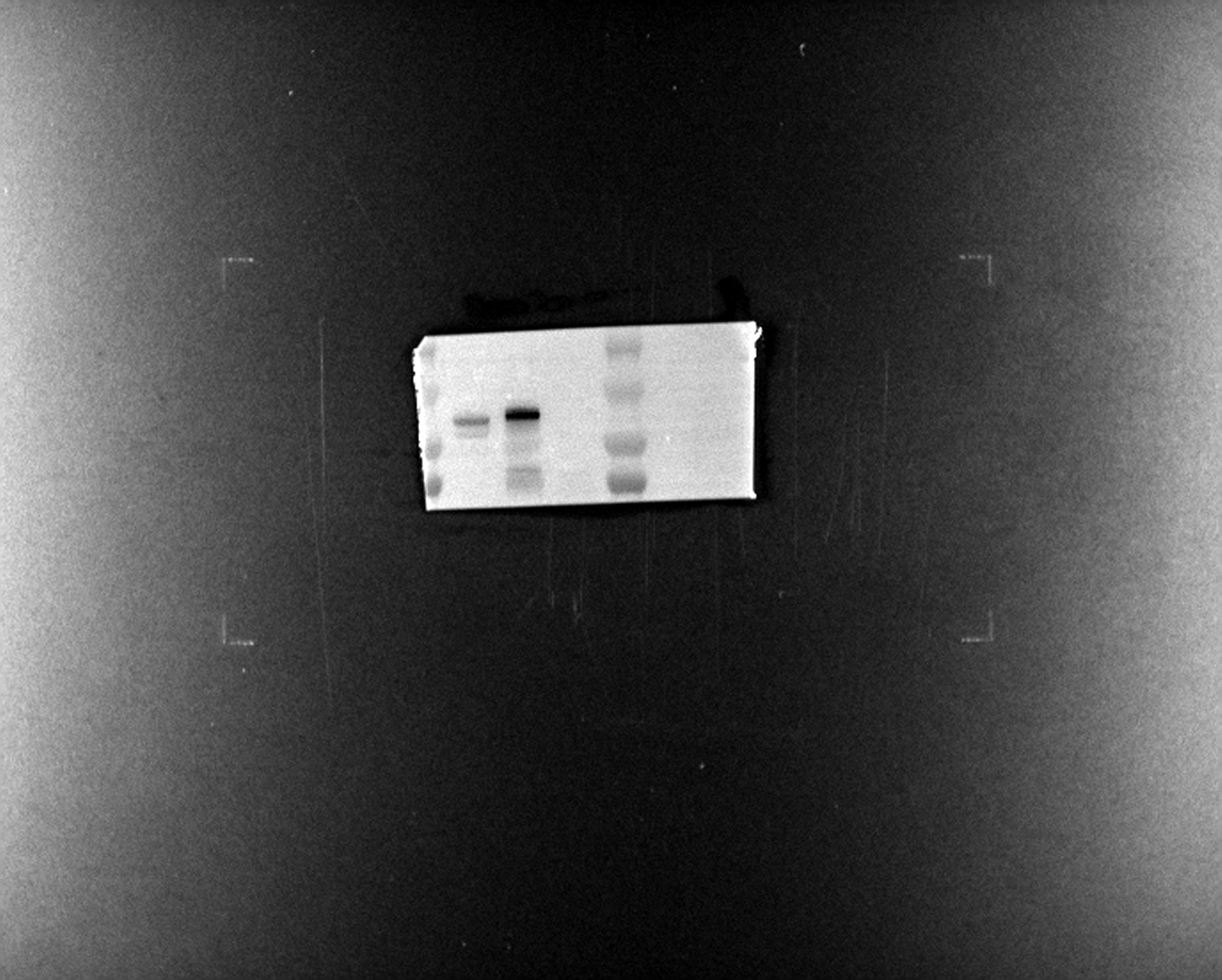

Supplement: Supplemental Information 4 [file peerj-11-15828-s004.zip › The raw data of Western blot/figure3c-bag3-upper.Tif]

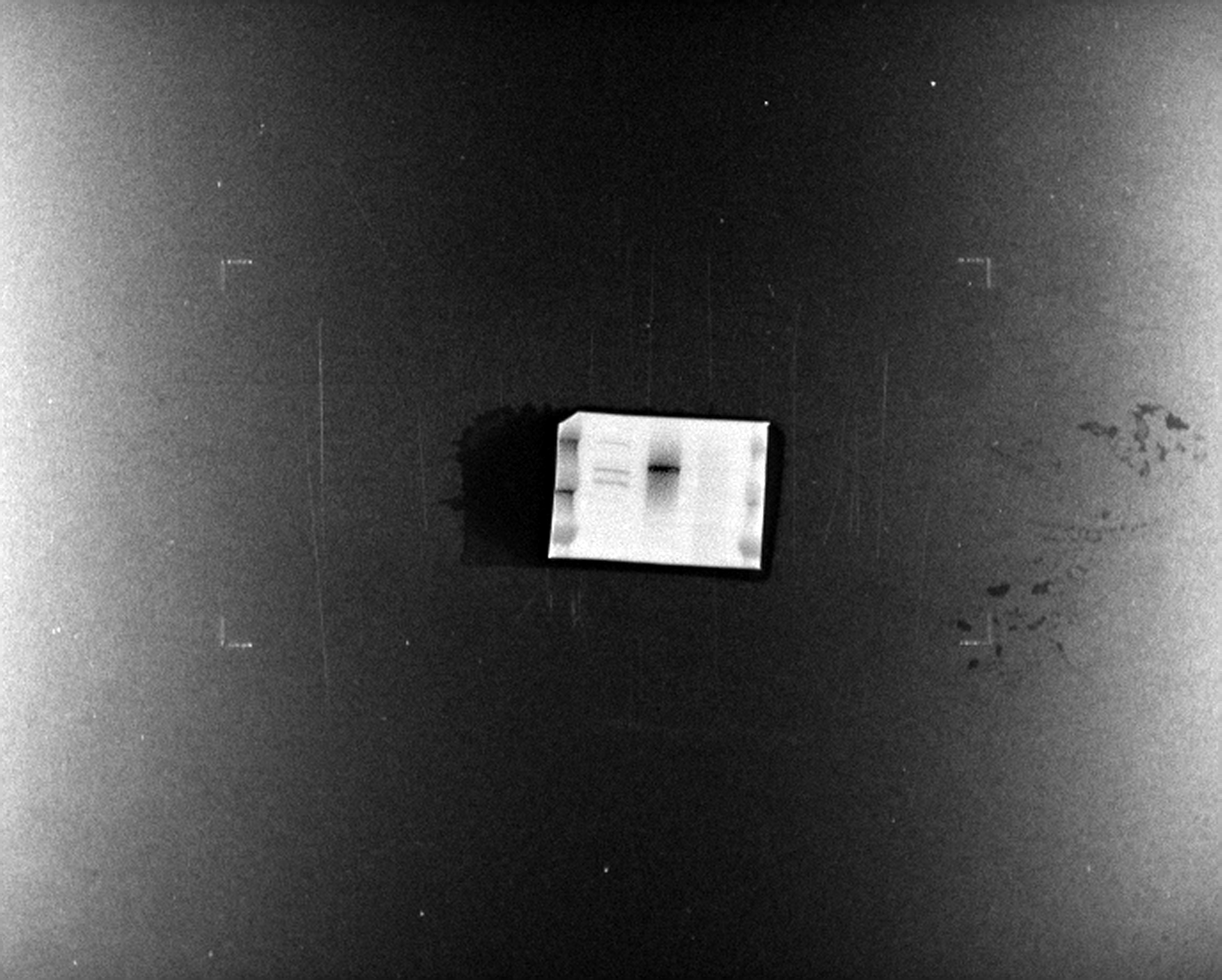

Supplement: Supplemental Information 4 [file peerj-11-15828-s004.zip › The raw data of Western blot/figure3c-ints7-down.Tif]

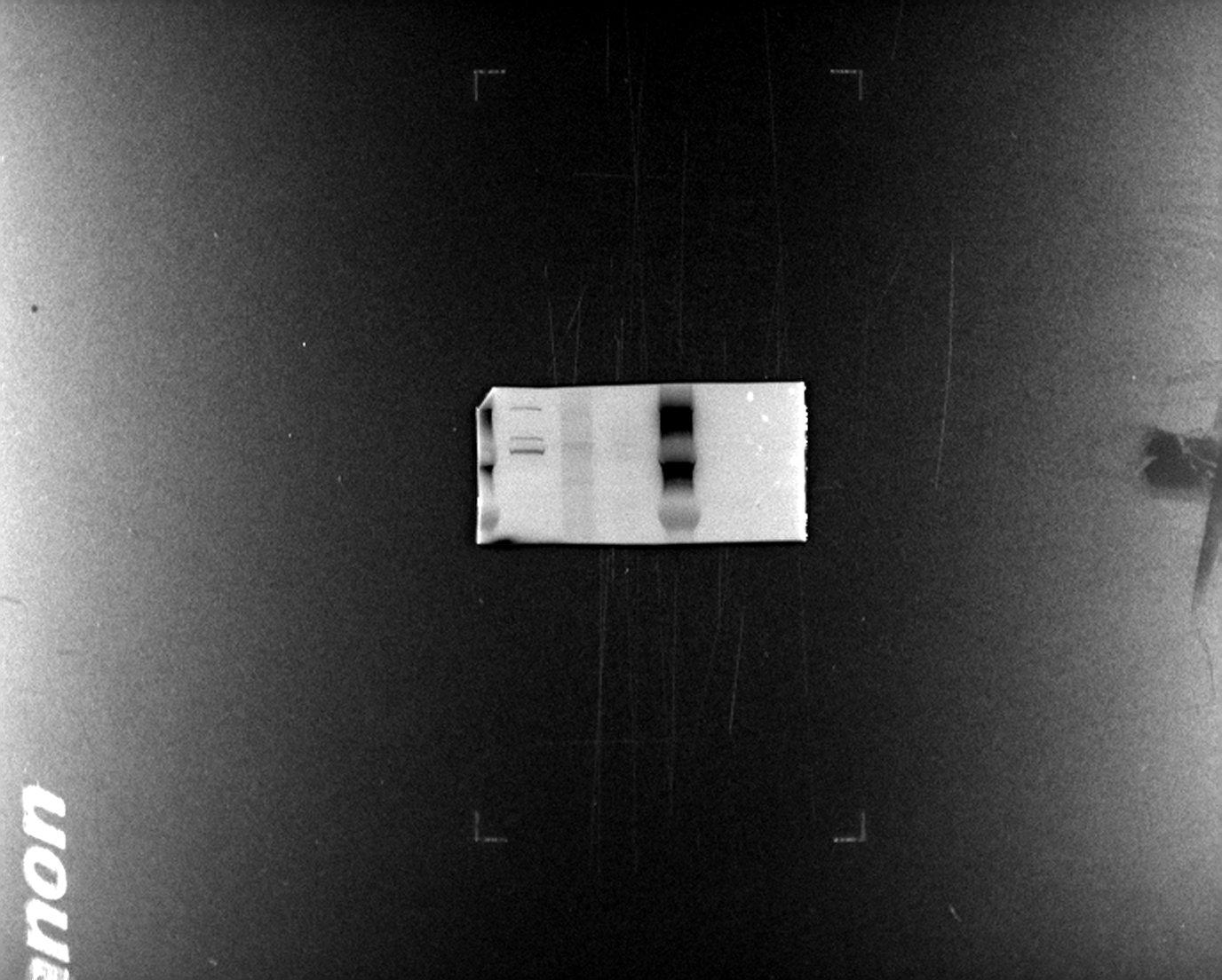

Supplement: Supplemental Information 4 [file peerj-11-15828-s004.zip › The raw data of Western blot/figure3c-ints7-upper.Tif]

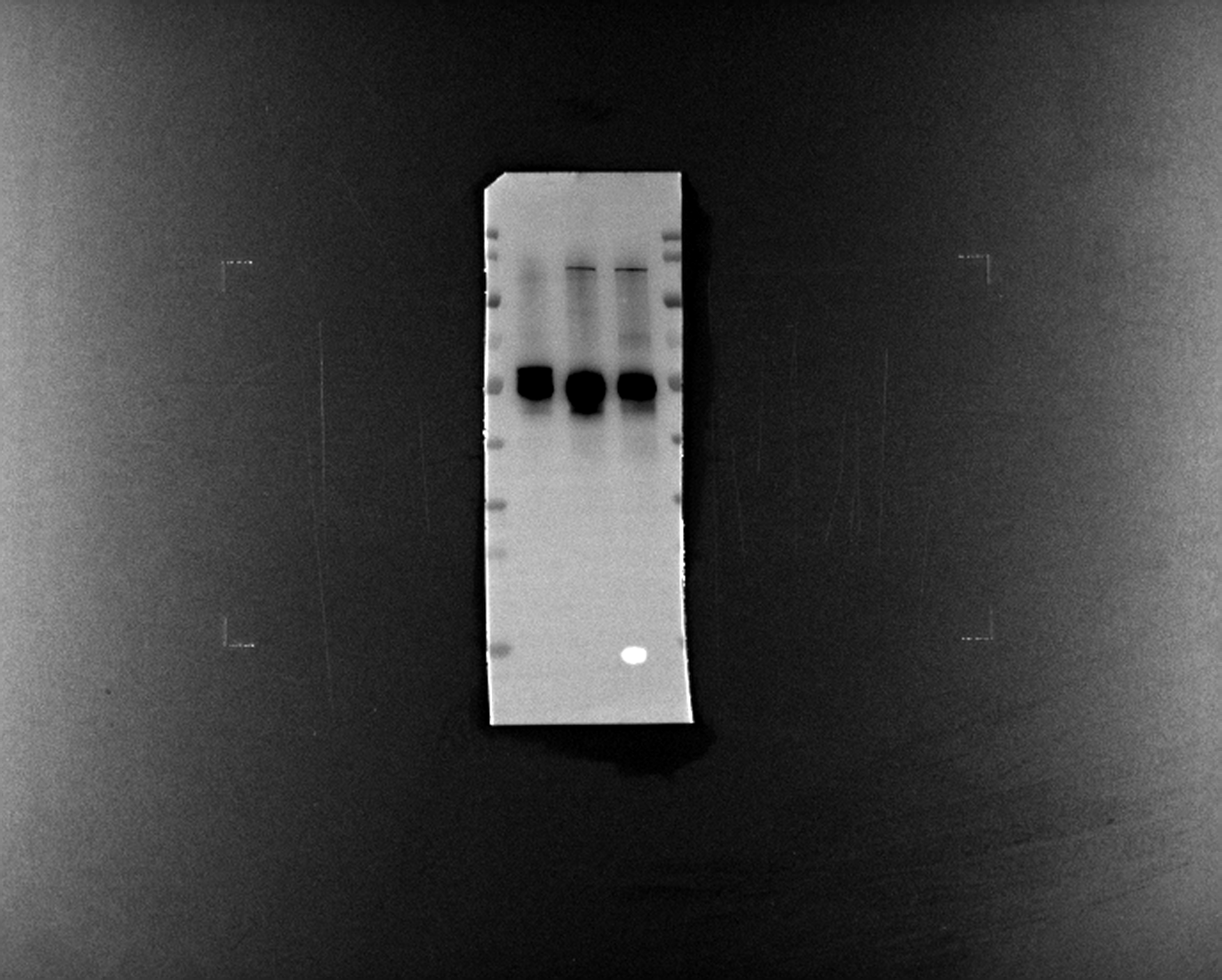

Supplement: Supplemental Information 4 [file peerj-11-15828-s004.zip › The raw data of Western blot/figure3h-ints7.Tif]

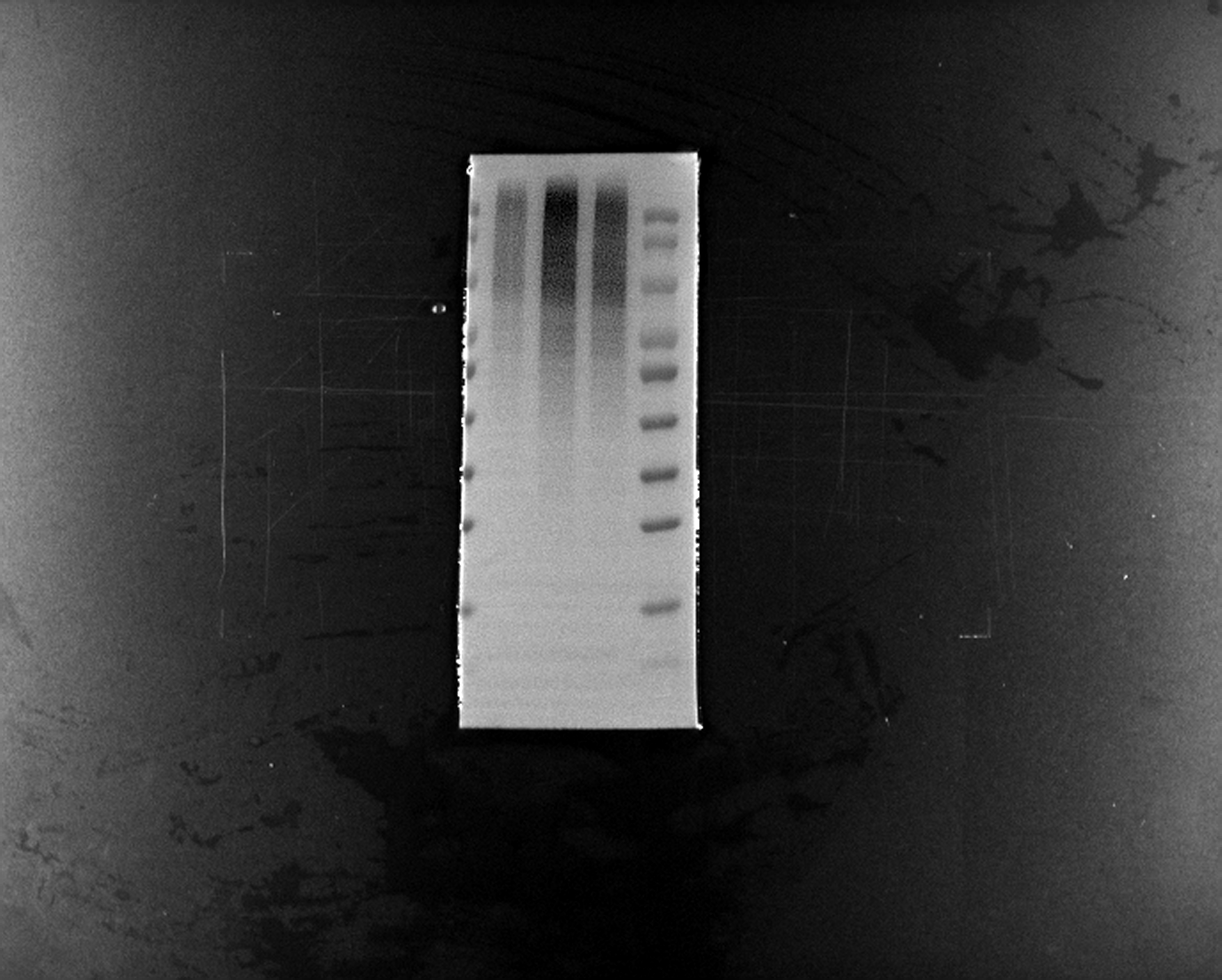

Supplement: Supplemental Information 4 [file peerj-11-15828-s004.zip › The raw data of Western blot/figure3h-ub.Tif]

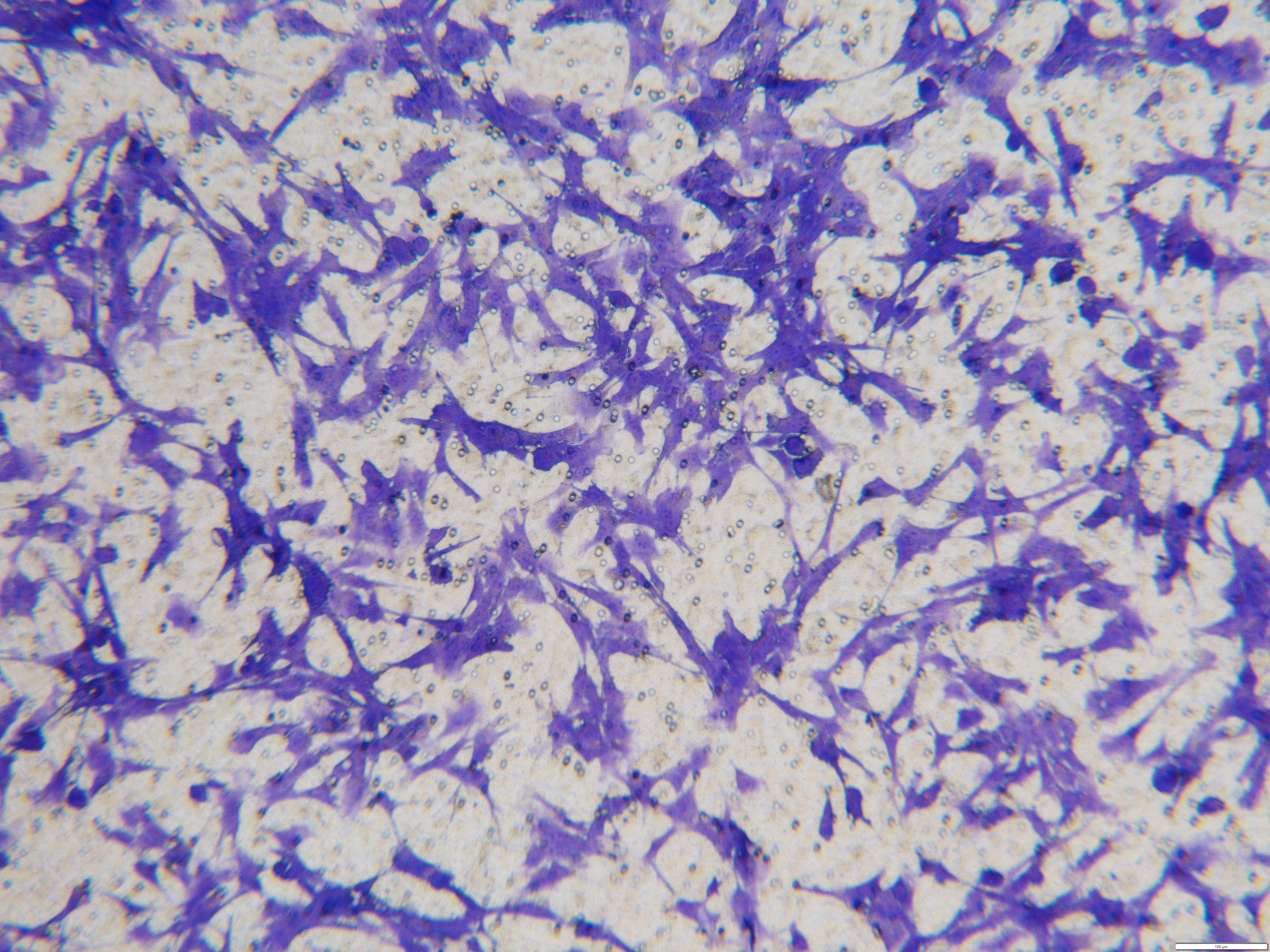

Supplement: Supplemental Information 5 [file peerj-11-15828-s005.zip › The raw data of transwell in figure 1and 2/figure1g/nc.jpg]

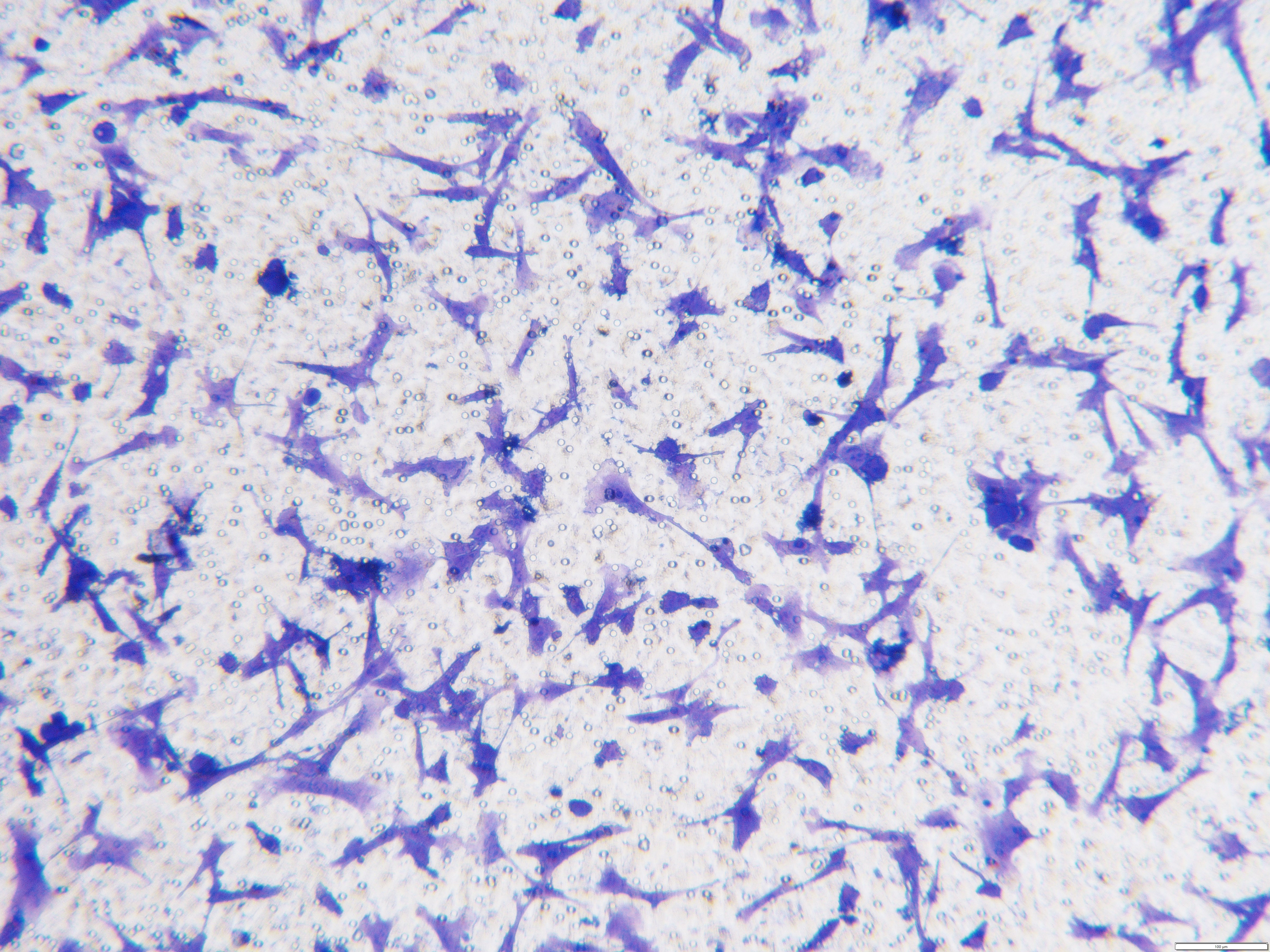

Supplement: Supplemental Information 5 [file peerj-11-15828-s005.zip › The raw data of transwell in figure 1and 2/figure1g/si-1.jpg]

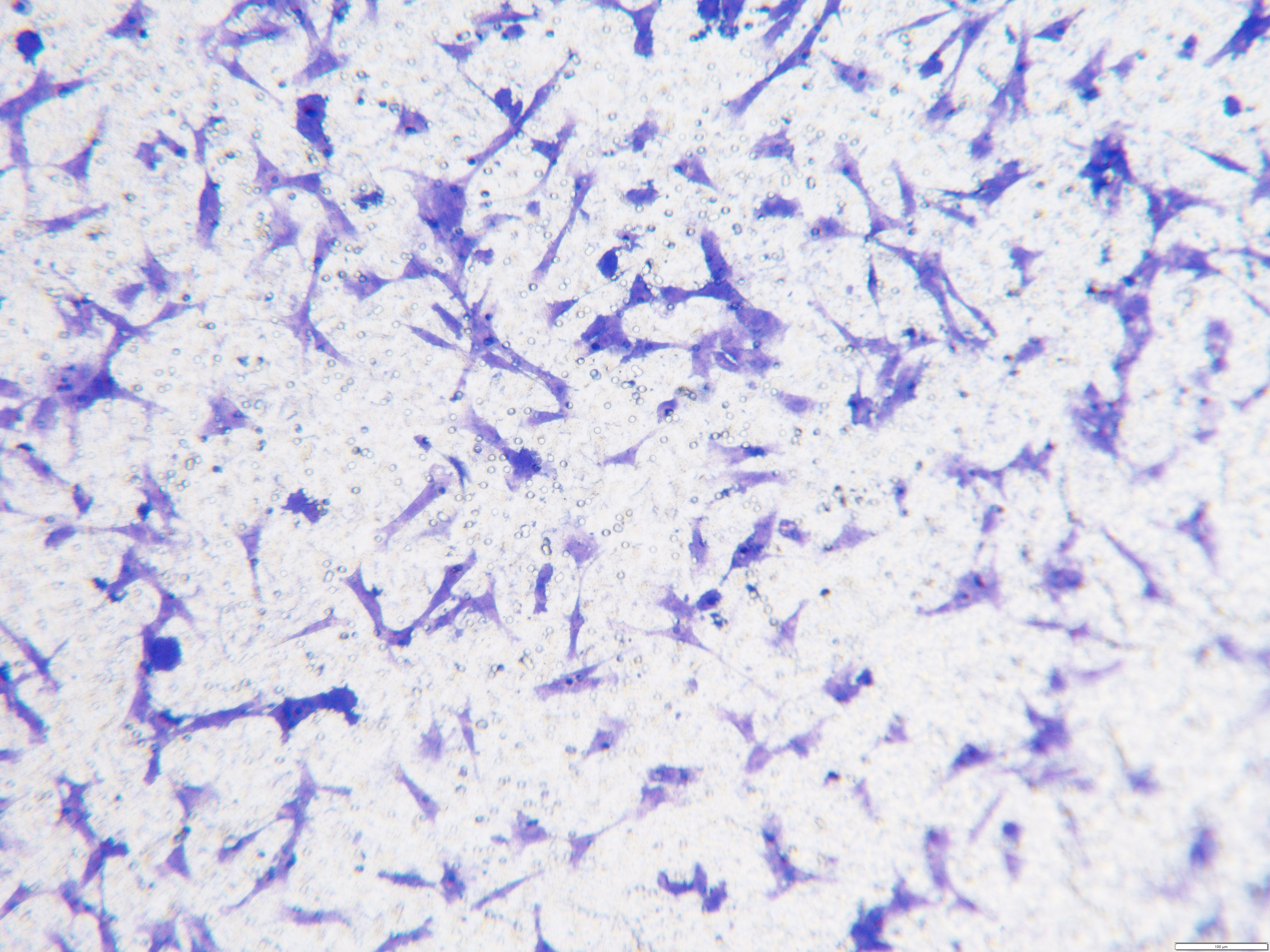

Supplement: Supplemental Information 5 [file peerj-11-15828-s005.zip › The raw data of transwell in figure 1and 2/figure1g/si-2.jpg]

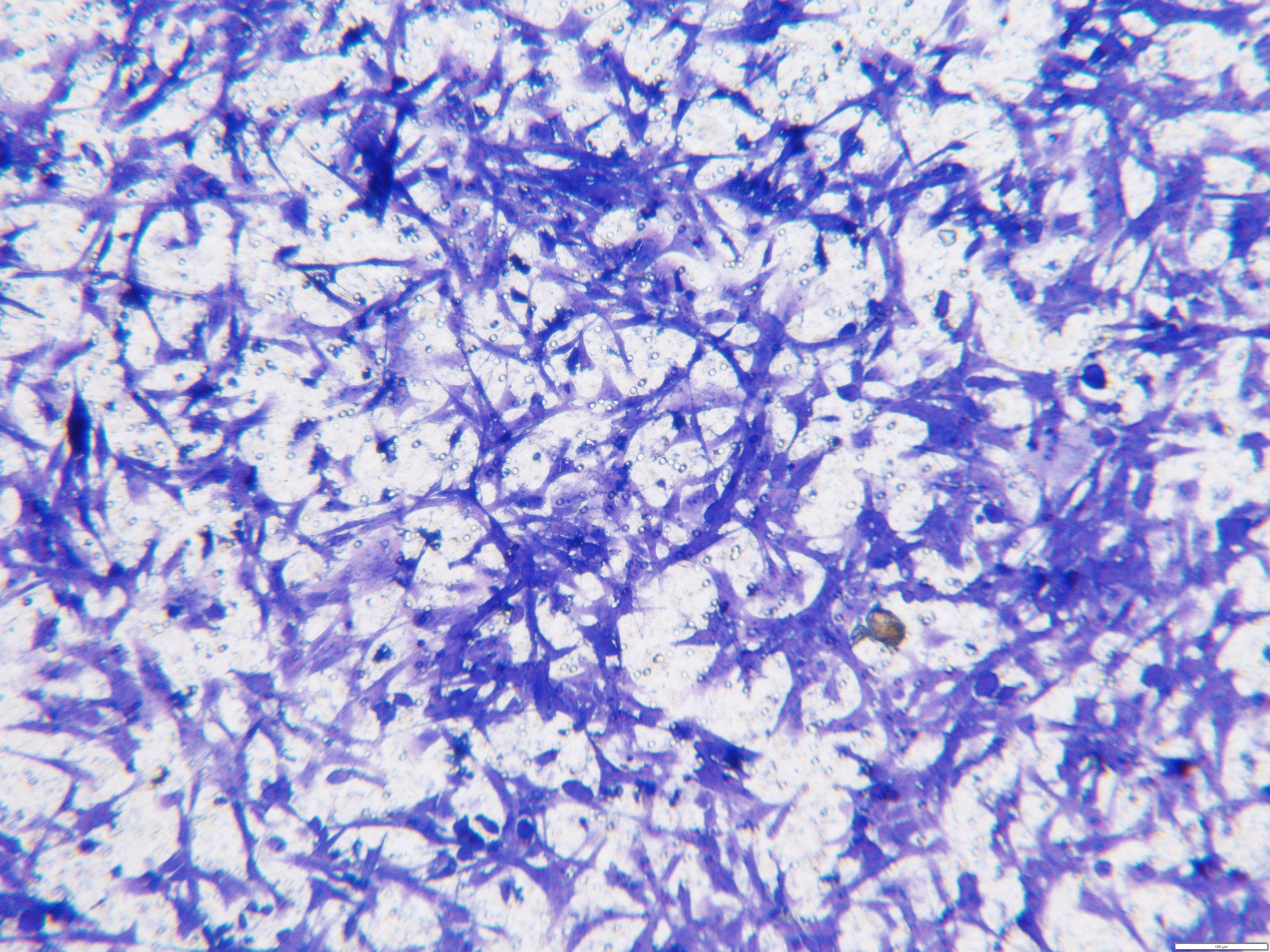

Supplement: Supplemental Information 5 [file peerj-11-15828-s005.zip › The raw data of transwell in figure 1and 2/figure2f/bag3.jpg]

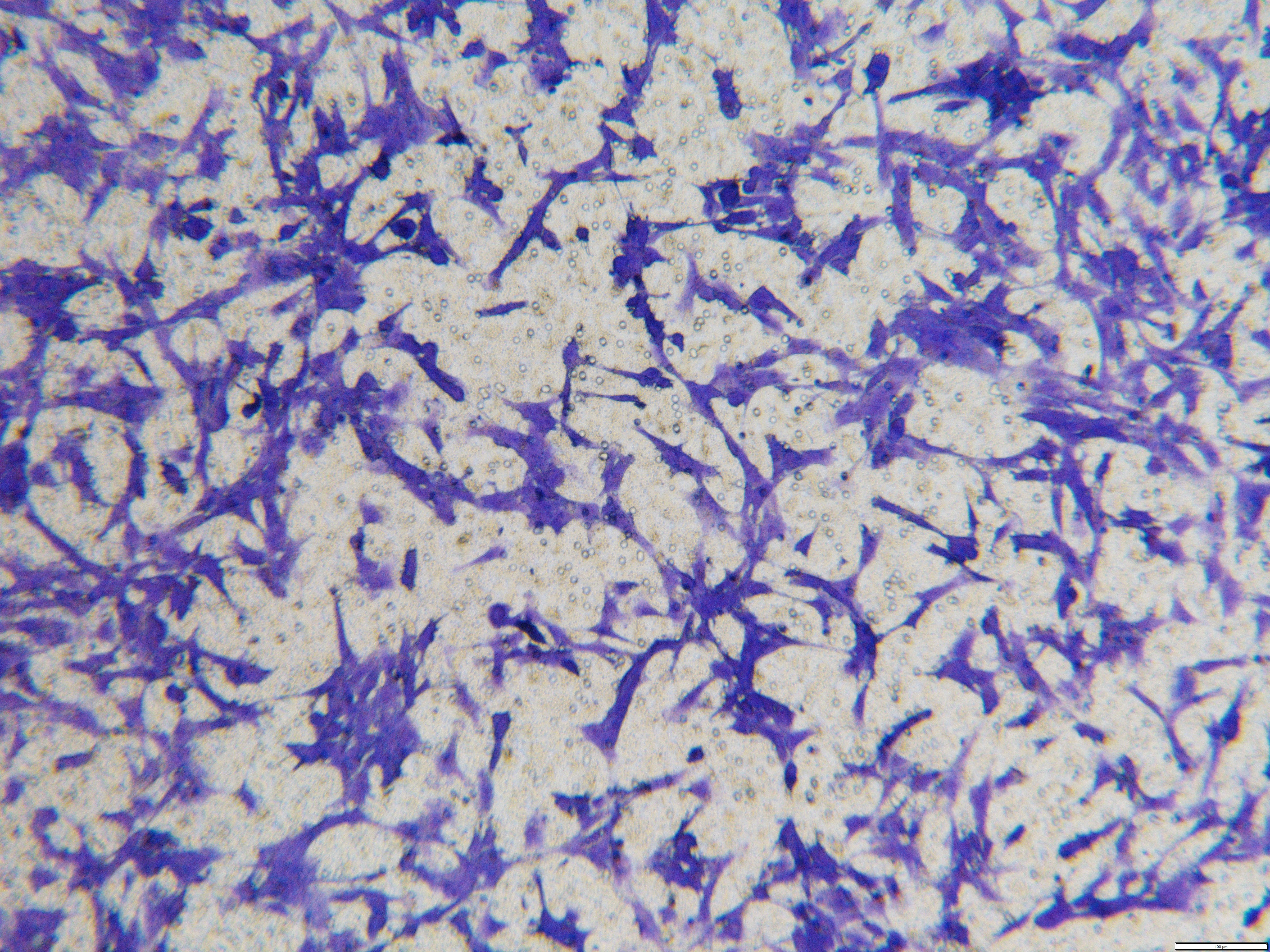

Supplement: Supplemental Information 5 [file peerj-11-15828-s005.zip › The raw data of transwell in figure 1and 2/figure2f/nc.jpg]

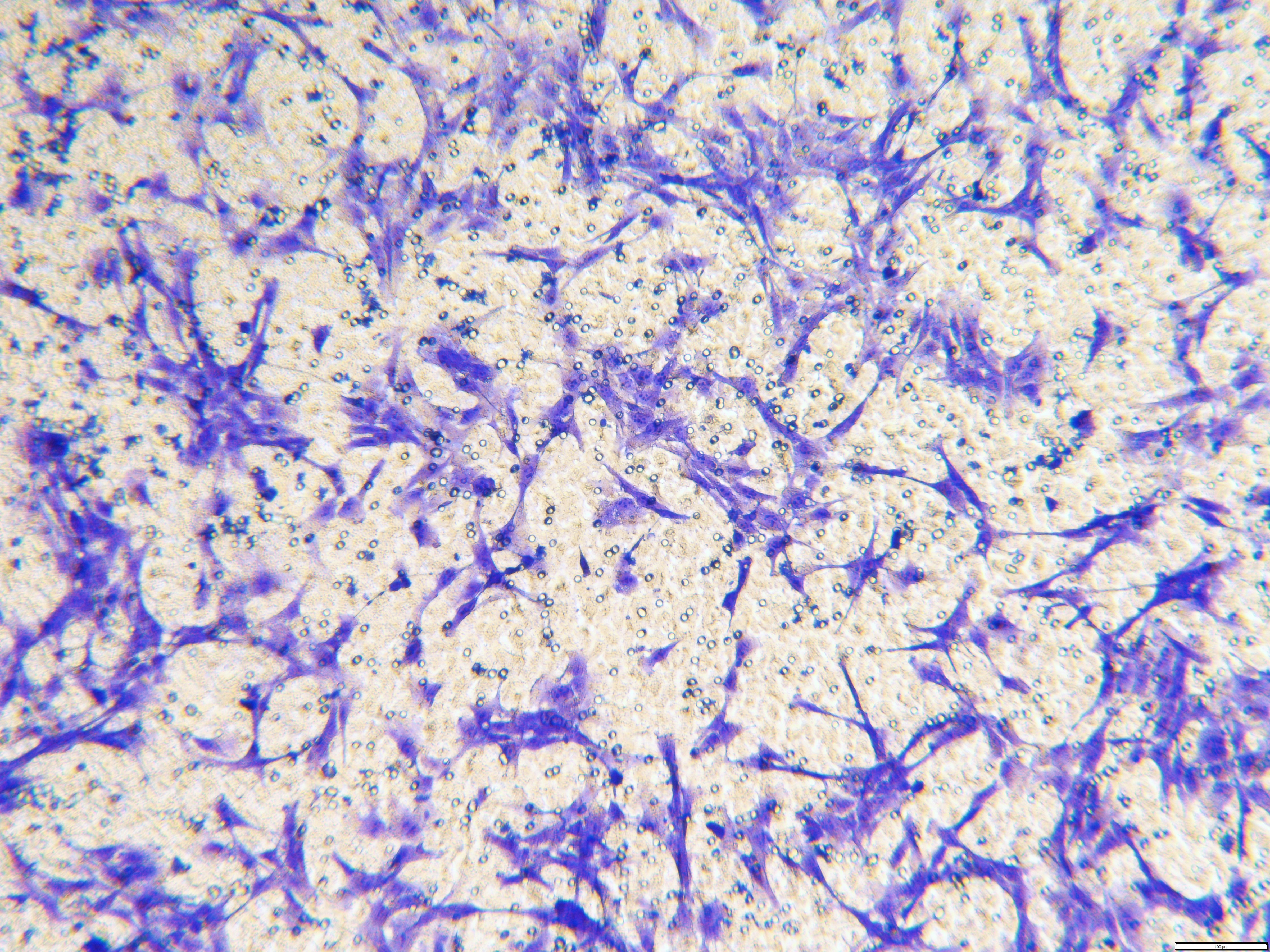

Supplement: Figure S5 [file peerj-11-15828-s006.zip › The raw data of transwell in figure 5/nc.jpg]

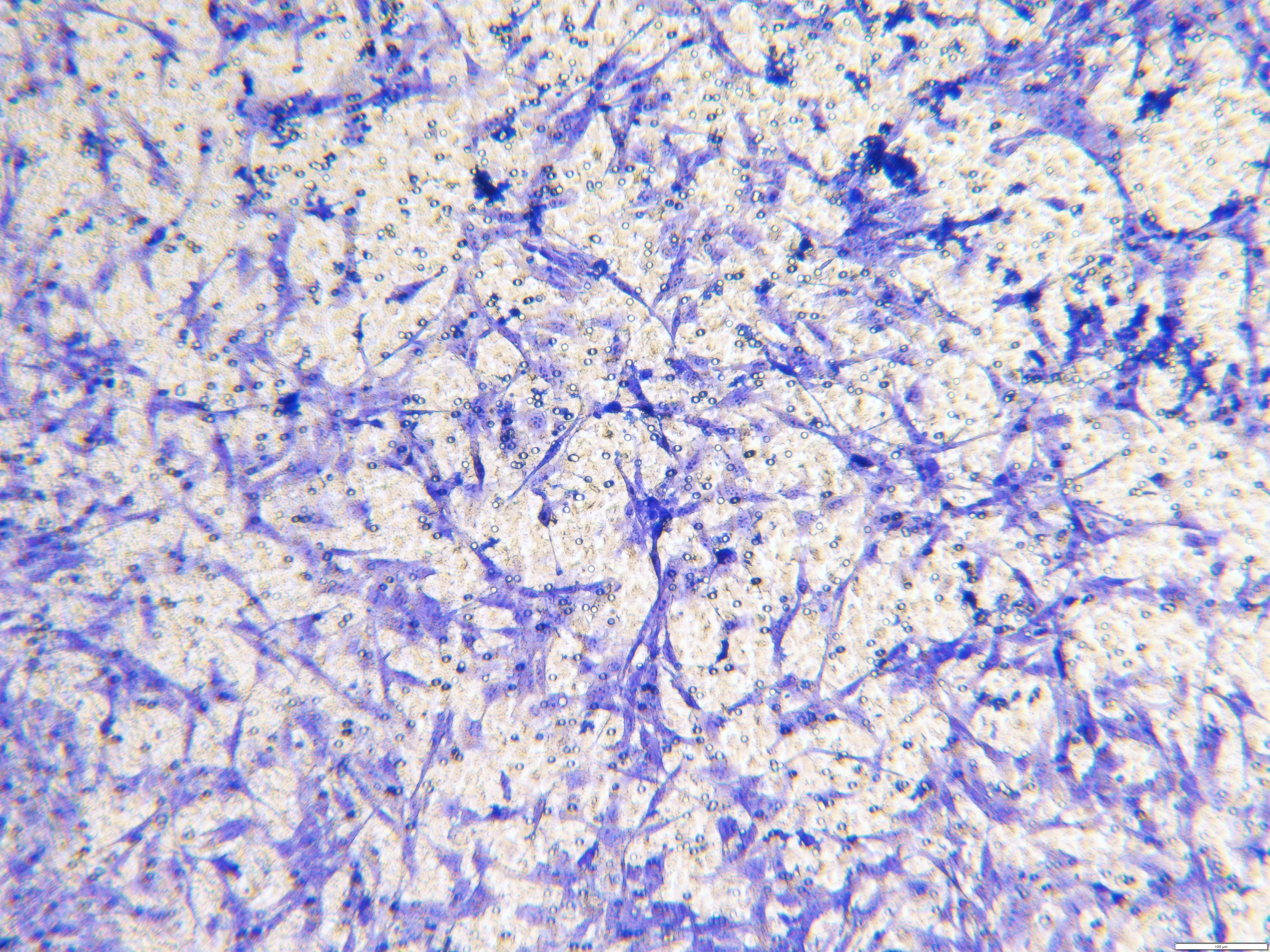

Supplement: Figure S5 [file peerj-11-15828-s006.zip › The raw data of transwell in figure 5/si-bag3+ints7.jpg]

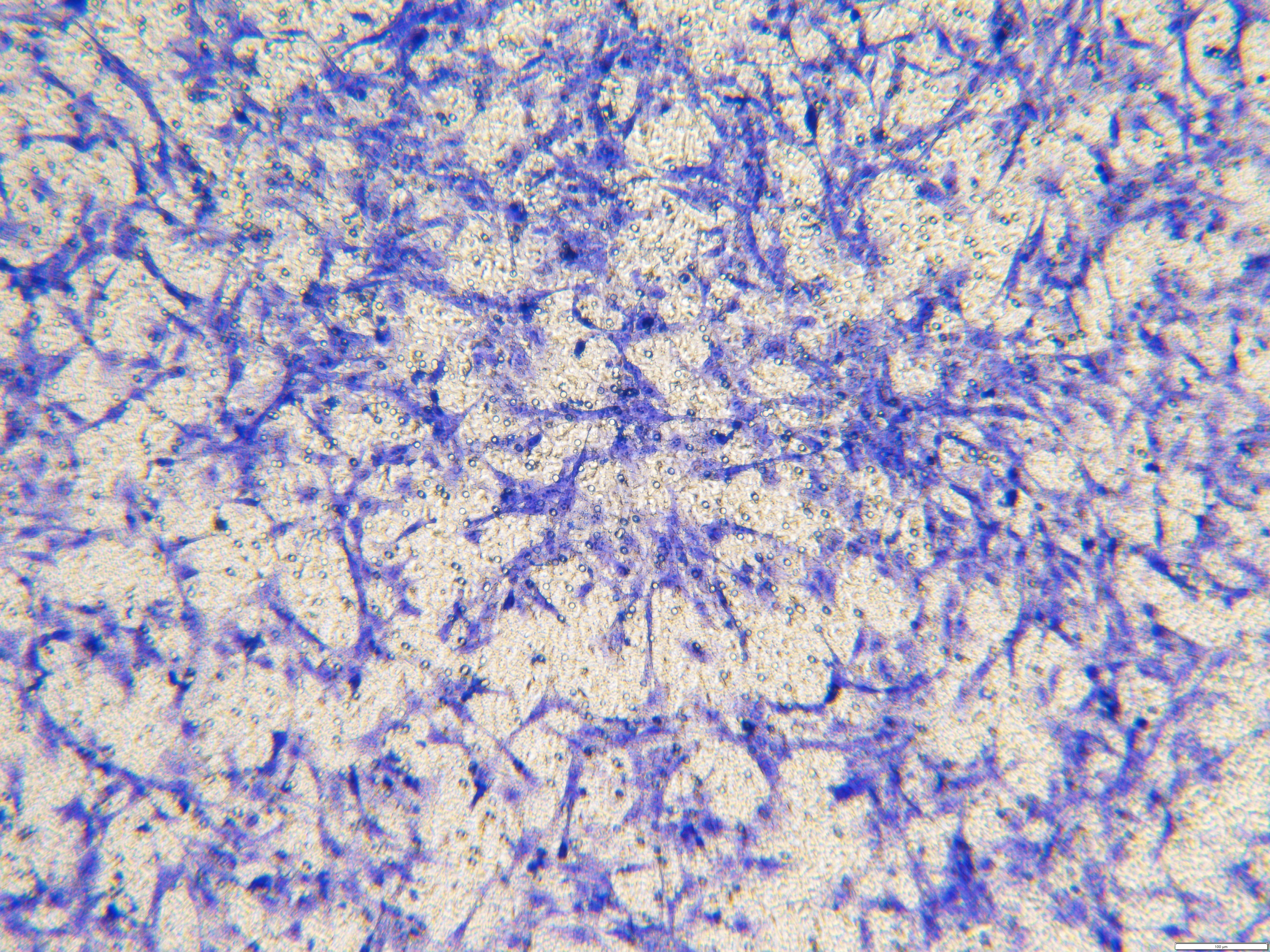

Supplement: Figure S5 [file peerj-11-15828-s006.zip › The raw data of transwell in figure 5/si-bag3+nac.jpg]

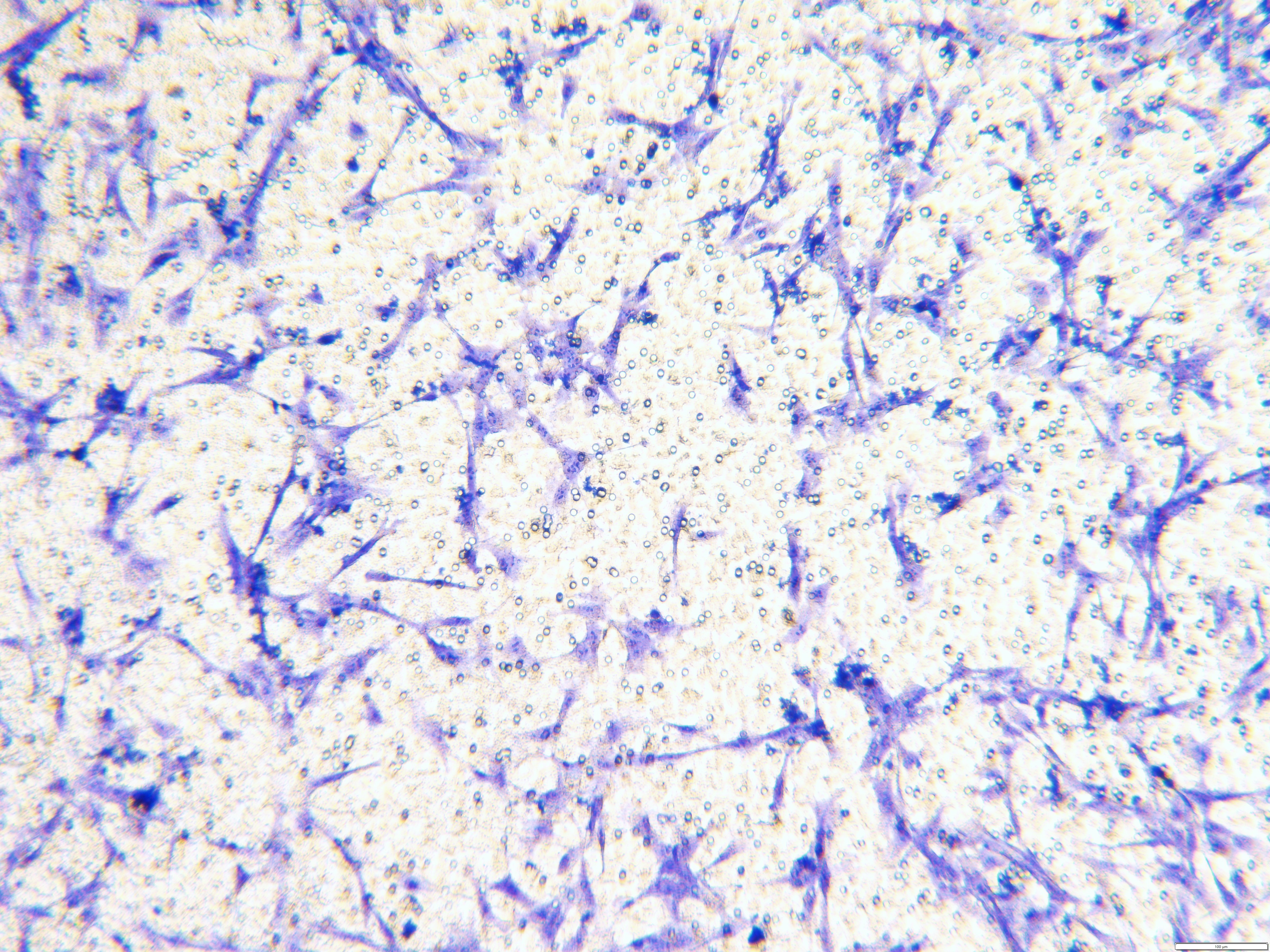

Supplement: Figure S5 [file peerj-11-15828-s006.zip › The raw data of transwell in figure 5/si-bag3.jpg]
