# Supplementary material for: BAG3 regulates bone marrow mesenchymal stem cell proliferation by targeting INTS7
Source: PeerJ. 2023 Aug 9;11:e15828. doi: 10.7717/peerj.15828 (PMC10422954; doi:10.7717/peerj.15828)
Supplement: Table S1 [file peerj-11-15828-s007.docx]

Supplementary data

**Supplementary Table 1: Binding residues**

| Receptor | Receptor  position | Receptor element | Ligand | Ligand  position | Ligand element | Distance (Å) |
| --- | --- | --- | --- | --- | --- | --- |
| INTS7 | 914 | ASN | BAG3 | 421 | PRO | 1.429 |
| INTS7 | 914 | ASN | BAG3 | 422 | PRO | 1.678 |
| INTS7 | 846 | ARG | BAG3 | 424 | HIS | 1.865 |
| INTS7 | 270 | ARG | BAG3 | 462 | TYR | 1.938 |
| INTS7 | 841 | PRO | BAG3 | 483 | GLN | 2.132 |
| INTS7 | 274 | GLN | BAG3 | 466 | GLU | 2.157 |
| INTS7 | 845 | ARG | BAG3 | 421 | PRO | 2.212 |
| INTS7 | 277 | LYS | BAG3 | 436 | LYS | 2.235 |
| INTS7 | 845 | ARG | BAG3 | 420 | THR | 2.259 |
| INTS7 | 846 | ARG | BAG3 | 427 | VAL | 2.316 |
| INTS7 | 270 | ARG | BAG3 | 458 | MET | 2.461 |
| INTS7 | 92 | LYS | BAG3 | 482 | ARG | 2.59 |
| INTS7 | 842 | GLY | BAG3 | 423 | LYS | 2.682 |
| INTS7 | 735 | THR | BAG3 | 417 | GLU | 2.687 |
| INTS7 | 282 | LYS | BAG3 | 429 | LYS | 2.713 |
| INTS7 | 697 | SER | BAG3 | 420 | THR | 2.723 |
| INTS7 | 844 | PHE | BAG3 | 420 | THR | 2.775 |
| INTS7 | 403 | PRO | BAG3 | 442 | GLN | 2.804 |
| INTS7 | 885 | ASN | BAG3 | 482 | ARG | 2.83 |
| INTS7 | 843 | LEU | BAG3 | 410 | PRO | 2.898 |
| INTS7 | 240 | LEU | BAG3 | 472 | SER | 2.914 |
| INTS7 | 274 | GLN | BAG3 | 465 | LYS | 3.043 |
| INTS7 | 914 | ASN | BAG3 | 420 | THR | 3.086 |
| INTS7 | 92 | LYS | BAG3 | 483 | GLN | 3.09 |
| INTS7 | 91 | GLU | BAG3 | 482 | ARG | 3.12 |
| INTS7 | 271 | LEU | BAG3 | 465 | LYS | 3.181 |
| INTS7 | 236 | HIS | BAG3 | 465 | LYS | 3.194 |
| INTS7 | 846 | ARG | BAG3 | 480 | ASP | 3.201 |
| INTS7 | 912 | ASP | BAG3 | 421 | PRO | 3.246 |
| INTS7 | 846 | ARG | BAG3 | 423 | LYS | 3.322 |
| INTS7 | 92 | LYS | BAG3 | 486 | ARG | 3.345 |
| INTS7 | 236 | HIS | BAG3 | 468 | LEU | 3.384 |
| INTS7 | 916 | ILE | BAG3 | 419 | GLU | 3.393 |
| INTS7 | 844 | PHE | BAG3 | 421 | PRO | 3.408 |
| INTS7 | 840 | LYS | BAG3 | 408 | ALA | 3.442 |
| INTS7 | 886 | ASP | BAG3 | 479 | ALA | 3.491 |
| INTS7 | 232 | ILE | BAG3 | 465 | LYS | 3.515 |
| INTS7 | 202 | GLN | BAG3 | 472 | SER | 3.541 |
| INTS7 | 884 | HIS | BAG3 | 477 | GLY | 3.678 |
| INTS7 | 885 | ASN | BAG3 | 477 | GLY | 3.712 |
| INTS7 | 240 | LEU | BAG3 | 468 | LEU | 3.732 |
| INTS7 | 1 | MET | BAG3 | 441 | GLU | 3.738 |
| INTS7 | 267 | ALA | BAG3 | 465 | LYS | 3.856 |
| INTS7 | 914 | ASN | BAG3 | 419 | GLU | 3.858 |
| INTS7 | 704 | GLU | BAG3 | 419 | GLU | 3.901 |
| INTS7 | 232 | ILE | BAG3 | 461 | GLU | 3.949 |
| INTS7 | 236 | HIS | BAG3 | 469 | ALA | 3.994 |
| INTS7 | 689 | TYR | BAG3 | 419 | GLU | 4.005 |
| INTS7 | 735 | THR | BAG3 | 418 | ALA | 4.008 |
| INTS7 | 202 | GLN | BAG3 | 468 | LEU | 4.024 |
| INTS7 | 404 | GLY | BAG3 | 442 | GLN | 4.038 |
| INTS7 | 916 | ILE | BAG3 | 418 | ALA | 4.067 |
| INTS7 | 274 | GLN | BAG3 | 462 | TYR | 4.085 |
| INTS7 | 240 | LEU | BAG3 | 469 | ALA | 4.094 |
| INTS7 | 885 | ASN | BAG3 | 479 | ALA | 4.107 |
| INTS7 | 1 | MET | BAG3 | 491 | LYS | 4.142 |
| INTS7 | 95 | GLU | BAG3 | 482 | ARG | 4.146 |
| INTS7 | 884 | HIS | BAG3 | 476 | GLU | 4.149 |
| INTS7 | 885 | ASN | BAG3 | 478 | ARG | 4.181 |
| INTS7 | 700 | LEU | BAG3 | 420 | THR | 4.202 |
| INTS7 | 843 | LEU | BAG3 | 408 | ALA | 4.213 |
| INTS7 | 701 | ARG | BAG3 | 419 | GLU | 4.255 |
| INTS7 | 281 | SER | BAG3 | 432 | ALA | 4.266 |
| INTS7 | 267 | ALA | BAG3 | 461 | GLU | 4.278 |
| INTS7 | 270 | ARG | BAG3 | 454 | LYS | 4.302 |
| INTS7 | 92 | LYS | BAG3 | 479 | ALA | 4.312 |
| INTS7 | 199 | PRO | BAG3 | 468 | LEU | 4.333 |
| INTS7 | 95 | GLU | BAG3 | 477 | GLY | 4.334 |
| INTS7 | 846 | ARG | BAG3 | 481 | VAL | 4.334 |
| INTS7 | 844 | PHE | BAG3 | 410 | PRO | 4.35 |
| INTS7 | 843 | LEU | BAG3 | 409 | GLU | 4.367 |
| INTS7 | 843 | LEU | BAG3 | 423 | LYS | 4.393 |
| INTS7 | 735 | THR | BAG3 | 416 | GLY | 4.416 |
| INTS7 | 733 | GLY | BAG3 | 418 | ALA | 4.422 |
| INTS7 | 1 | MET | BAG3 | 406 | ALA | 4.427 |
| INTS7 | 1 | MET | BAG3 | 495 | ILE | 4.43 |
| INTS7 | 913 | ALA | BAG3 | 421 | PRO | 4.487 |
| INTS7 | 1 | MET | BAG3 | 438 | GLN | 4.487 |
| INTS7 | 4 | ASN | BAG3 | 406 | ALA | 4.498 |
| INTS7 | 695 | ALA | BAG3 | 420 | THR | 4.534 |
| INTS7 | 95 | GLU | BAG3 | 476 | GLU | 4.534 |
| INTS7 | 846 | ARG | BAG3 | 421 | PRO | 4.538 |
| INTS7 | 270 | ARG | BAG3 | 465 | LYS | 4.565 |
| INTS7 | 274 | GLN | BAG3 | 436 | LYS | 4.569 |
| INTS7 | 278 | LEU | BAG3 | 436 | LYS | 4.614 |
| INTS7 | 278 | LEU | BAG3 | 433 | ILE | 4.64 |
| INTS7 | 696 | ASP | BAG3 | 420 | THR | 4.646 |
| INTS7 | 883 | PRO | BAG3 | 477 | GLY | 4.675 |
| INTS7 | 733 | GLY | BAG3 | 419 | GLU | 4.676 |
| INTS7 | 1 | MET | BAG3 | 407 | PRO | 4.694 |
| INTS7 | 1 | MET | BAG3 | 498 | LYS | 4.704 |
| INTS7 | 282 | LYS | BAG3 | 473 | VAL | 4.758 |
| INTS7 | 282 | LYS | BAG3 | 472 | SER | 4.826 |
| INTS7 | 202 | GLN | BAG3 | 471 | ASP | 4.878 |
| INTS7 | 91 | GLU | BAG3 | 485 | ARG | 4.88 |
| INTS7 | 846 | ARG | BAG3 | 425 | PRO | 4.906 |
| INTS7 | 844 | PHE | BAG3 | 422 | PRO | 4.908 |
| INTS7 | 913 | ALA | BAG3 | 422 | PRO | 4.962 |
| INTS7 | 1 | MET | BAG3 | 494 | THR | 4.97 |
| INTS7 | 841 | PRO | BAG3 | 423 | LYS | 4.982 |
| INTS7 | 1 | MET | BAG3 | 445 | ASP | 4.995 |
